# Human iPSC-derived mesenchymal progenitor cell sheets promote tissue formation after xenotransplantation

**DOI:** 10.1016/j.isci.2026.117128

**Published:** 2026-08-11

**Authors:** Shu Obana, Junta Minato, Saki Takahashi, Shoko Itakura, Makiya Nishikawa, Kosuke Kusamori

**Affiliations:** 1 Laboratory of Cellular Drug Discovery and Development, Faculty of Pharmaceutical Sciences, Tokyo University of Science, 6-3-1 Niijuku, Katsushika, Tokyo 125-8585, Japan; 2 Laboratory of Biopharmaceutics, Faculty of Pharmaceutical Sciences, Tokyo University of Science, 6-3-1 Niijuku, Katsushika, Tokyo 125-8585, Japan; 3 Research Institute for Science and Technology (RIST), Tokyo University of Science, 2641 Yamazaki, Noda, Chiba 278-8510, Japan

**Keywords:** human iPSC-derived mesenchymal progenitor cells, cell sheet engineering, xenotransplantation, immunomodulation, tissue formation, stromal framework, lymph node transplantation, regenerative medicine

## Abstract

Mesenchymal stromal cell (MSC) sheets are a promising platform for regenerative therapy, but their *in vivo* survival and capacity to form tissue-like structures remain poorly understood. Here, we investigated whether sheets derived from human induced pluripotent stem cell (iPSC)-derived mesenchymal progenitor cells (hiPS-MPCs) can survive and form tissue-like structures after xenotransplantation. hiPS-MPCs were differentiated from human iPSCs, validated by surface marker expression and multilineage differentiation potential, and assembled into intact cell sheets. Mouse MSC, fibroblast, and hiPS-MPC sheets were transplanted subcutaneously into immunocompetent mice. By day 7, mouse MSC and hiPS-MPC sheets formed organized vascularized tissue-like structures, whereas fibroblast sheets frequently induced inflammatory responses and showed limited persistence. Notably, hiPS-MPC-derived tissues exhibited reduced immune cell infiltration and higher expression of anti-inflammatory cytokines than fibroblast tissues. These findings demonstrate that hiPS-MPC sheets generate vascularized tissues *in vivo* and support survival across species, highlighting their potential as a scalable regenerative therapy platform.

## Highlights

• Human iPSC-derived MPC sheets survive after xenotransplantation
• Cell sheets establish an immunoregulatory microenvironment *in vivo*
• Immunomodulation supports long-term vascularized tissue formation
• hiPS-MPC-based sheets improve lymphatic drainage in mice

## Graphical abstract

**Figure:**
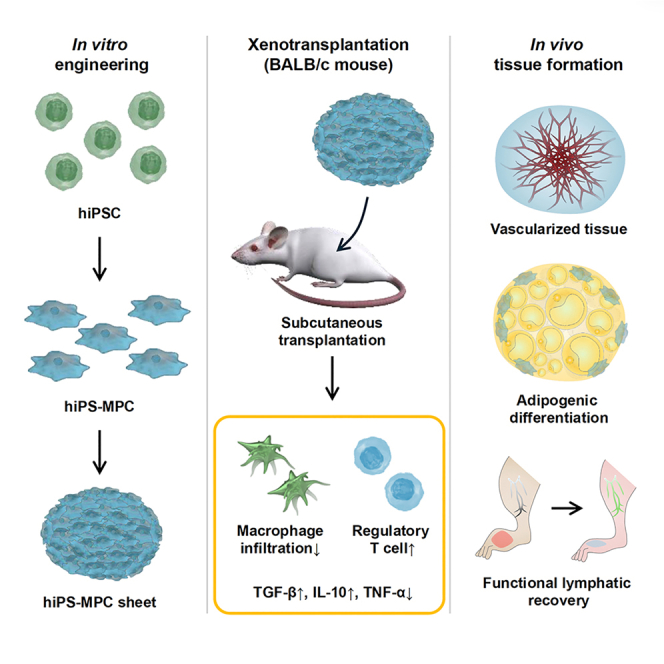

## Teaser

Biological sciences; Cell biology; Immunity; Stem cells research

## Introduction

Mesenchymal stromal cells (MSCs) are multipotent cells that have attracted considerable attention in regenerative medicine owing to their capacity for self-renewal, multilineage differentiation, and potent paracrine activity.^1^^,^^2^ Rather than functioning solely through direct differentiation, MSCs exert therapeutic effects primarily through the secretion of bioactive molecules that regulate inflammation, angiogenesis, and tissue repair. These paracrine factors include immunomodulatory mediators such as indoleamine 2,3-dioxygenase, prostaglandin E2, and transforming growth factor β (TGF-β), as well as trophic and pro-angiogenic molecules including vascular endothelial growth factor.^3^ Through these mechanisms, MSCs can suppress excessive immune responses and promote tissue regeneration in damaged or inflamed environments. Consequently, MSC-based therapies have been widely investigated for the treatment of inflammatory and degenerative diseases, and several MSC products have been clinically applied for conditions such as graft-versus-host disease.^4^ MSCs can be isolated from a variety of adult tissues, including bone marrow, adipose tissue, and umbilical cord. However, their clinical translation remains limited by several practical constraints. In particular, donor-to-donor variability, restricted proliferative capacity during *in vitro* expansion, and the invasive procedures required for cell procurement can compromise scalability and reproducibility.^5^ Induced pluripotent stem cell (iPSC)-derived mesenchymal progenitor cells (iPS-MPCs) have emerged as a promising alternative cell source to address these limitations. iPS-MPCs can be generated from pluripotent stem cells with minimal donor dependence, extensively expanded while maintaining stable characteristics, and produced with high batch-to-batch consistency, thereby offering a scalable and standardized platform for cell-based therapy.^6^ Nevertheless, similar to primary MSCs, transplanted iPS-MPCs frequently experience substantial cellular stress after administration, and many cells undergo apoptosis shortly after injection, resulting in limited survival and reduced therapeutic persistence *in vivo*.

Cell sheet engineering has emerged as an effective strategy for enhancing the retention, survival, and therapeutic efficacy of transplanted cells.^7^ Unlike conventional transplantation approaches, in which cells are delivered as suspensions and often undergo rapid anoikis or mechanical loss after injection, cell sheet technology enables the transplantation of intact cellular layers while preserving cell-cell junctions, extracellular matrix components, and endogenous signaling cues.^8^^,^^9^ This structural integrity allows grafted cells to adhere directly to host tissues without the need for artificial scaffolds. It helps maintain a supportive microenvironment that promotes cell survival and functional integration. In addition, the preserved extracellular matrix within the cell sheets facilitates early vascular infiltration and improves nutrient and oxygen supply after transplantation, thereby supporting the formation of organized tissue-like structures *in vivo*. Several studies have demonstrated that MSC-based cell sheets enhance cell engraftment and prolong cell persistence compared with dissociated cell suspensions, thereby improving outcomes in experimental models of tissue repair.^10^^,^^11^ Consistent with these findings, we previously reported that MSC sheets survived substantially longer than suspended MSCs and generated organized tissue-like structures following subcutaneous implantation in nude mice.^12^ However, these studies were performed in immunodeficient hosts that lack functional T cell-mediated immune responses. Consequently, it remains unclear whether similar tissue formation and graft persistence can be achieved in immunocompetent recipients, in whom immune surveillance and rejection mechanisms may critically influence graft survival. Moreover, it remains unclear whether xenogeneic cell sheets derived from pluripotent stem cells can modulate host immune responses sufficiently to enable stable tissue formation across species barriers.

In addition to their regenerative capacity, MSCs are widely recognized for their ability to modulate host immune responses. MSCs interact with innate and adaptive immune cells, including macrophages, dendritic cells, T lymphocytes, and natural killer cells, and thereby shape the inflammatory microenvironment after transplantation.^13^^,^^14^^,^^15^ Through the secretion of these soluble mediators, MSCs suppress excessive inflammatory activation and promote macrophage polarization toward anti-inflammatory phenotypes. These immunomodulatory properties are considered major contributors to the therapeutic benefits of MSC-based therapies, particularly in inflammatory and autoimmune diseases. Importantly, accumulating evidence suggests that MSCs may exhibit a degree of immune privilege, enabling them to persist transiently even in allogeneic or xenogeneic environments. However, the extent to which pluripotent stem cell-derived MSC-like cells retain their immunomodulatory properties, and in particular, whether iPSC-derived mesenchymal progenitor cells can regulate host immune responses sufficiently to support stable graft survival and tissue formation following xenotransplantation, remains unclear.

In this study, we investigated whether hiPSC-derived mesenchymal progenitor cell (hiPS-MPC) sheets can survive, integrate, and form tissue-like structures following xenotransplantation into immunocompetent mice. We found that hiPS-MPC sheets attenuated inflammatory responses, as evidenced by reduced macrophage infiltration and increased expression of anti-inflammatory cytokines, effects that persisted for prolonged periods *in vivo*. In contrast, mouse fibroblasts (NIH3T3) sheets exhibited increased macrophage infiltration and lower expression of anti-inflammatory cytokines. hiPS-MPC-derived tissues developed into organized tissue-like constructs exhibiting structural features resembling early stromal frameworks. In contrast, NIH3T3 sheets induced pro-inflammatory cytokine production and robust immune cell recruitment. Collectively, these findings demonstrate that hiPS-MPC sheets possess intrinsic immunomodulatory and pro-regenerative properties that support long-term graft survival and tissue formation in immunocompetent xenogeneic environments, highlighting their potential as a platform technology for regenerative medicine.

## Results

### Generation and characterization of hiPS-MPCs

**Figure 1:**
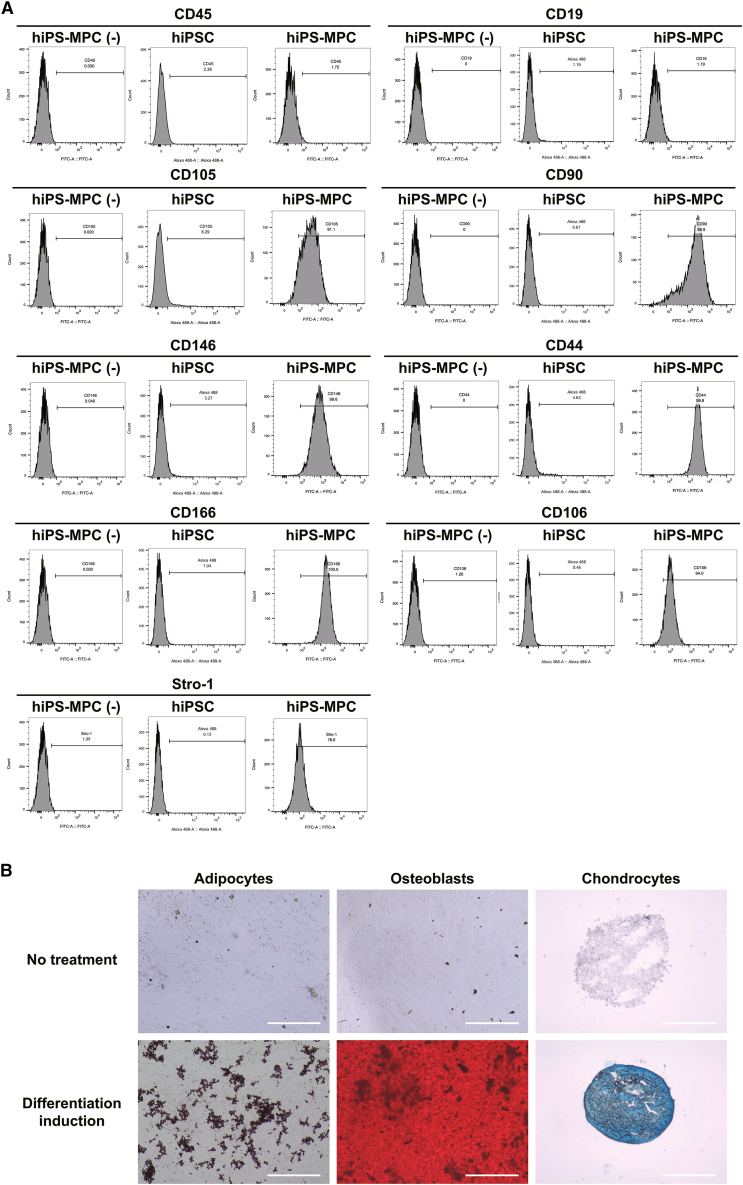
Characterization of hiPS-MPCs (A) Flow cytometric analysis demonstrates that hiPS-MPCs are negative for the hematopoietic markers CD45 and CD19 and positive for mesenchymal stromal cell (MSC) markers, including CD105, CD90, CD146, CD44, CD166, CD106, and Stro-1. hiPS-MPC (−) indicates unstained hiPS-MPCs used as a negative control to define background signal. (B) Representative images of hiPS-MPCs after multilineage differentiation into adipocytes, osteoblasts, and chondrocytes, as assessed using Oil Red O, Alizarin Red S, and Alcian blue staining, respectively. Scale bars, 100 μm.

hiPSCs were differentiated into mesenchymal progenitor cells (hiPS-MPCs) according to the manufacturer’s instructions using a commercially available mesenchymal progenitor differentiation kit (STEMdiff Mesenchymal Progenitor Kit, STEMCELL Technologies, Vancouver, Canada). The expression of MSC-associated surface markers in hiPS-MPCs was examined using flow cytometry. hiPS-MPCs were positive for typical MSC markers, including Stro-1, CD44, CD90, CD105, CD106, CD146, and CD166, whereas the hematopoietic markers CD19 and CD45 were not detected (Figure 1A). Next, we evaluated the multilineage differentiation potential of hiPS-MPCs. Upon induction with lineage-specific differentiation medium, hiPS-MPCs differentiated into adipocytes, osteoblasts, and chondrocytes. Adipogenic, osteogenic, and chondrogenic differentiation were confirmed using Oil Red O, Alizarin Red S, and Alcian blue staining, respectively (Figure 1B). These results confirmed that hiPSCs successfully differentiated into MSC-like progenitor cells, exhibiting characteristic surface marker expression and multilineage differentiation potential.

### Fabrication and characterization of hiPS-MPC sheets

**Figure 2:**
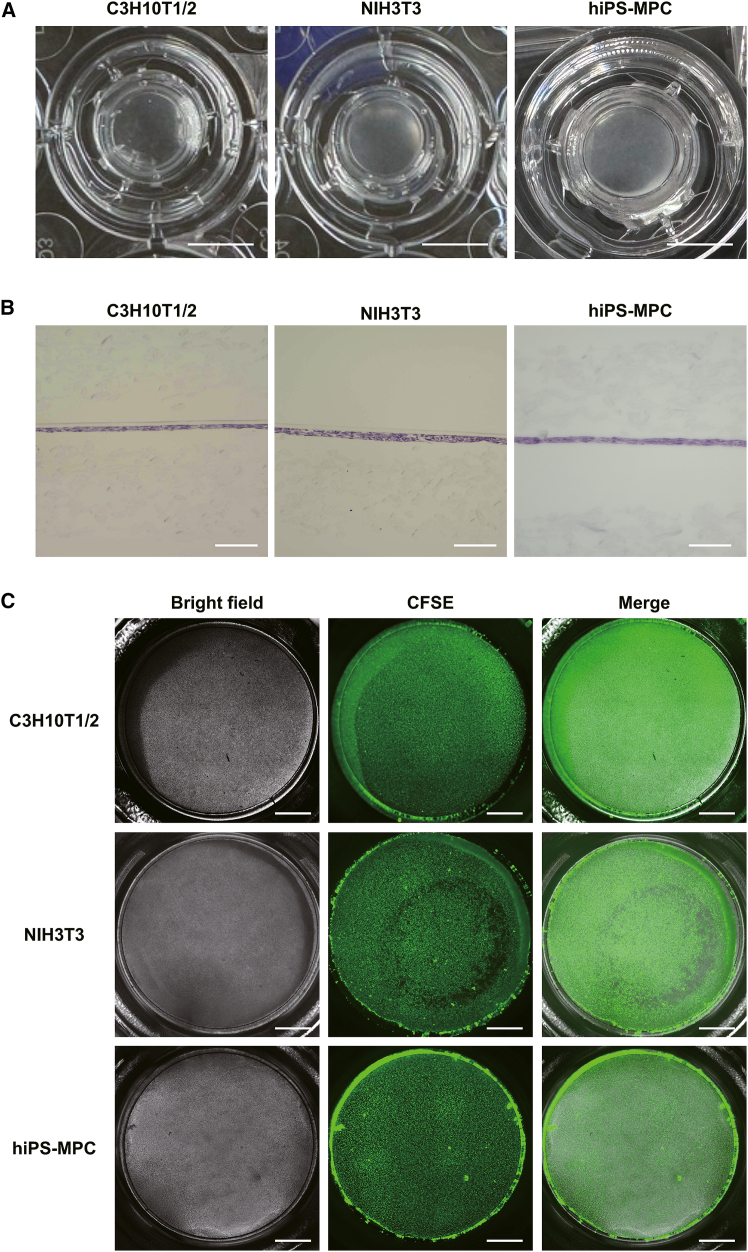
Fabrication of cell sheets (A) Representative macroscopic images of mouse mesenchymal stromal cell (C3H10T1/2), mouse fibroblast (NIH3T3), and human iPSC-derived mesenchymal progenitor cell (hiPS-MPC) sheets on day 5. Scale bars, 5 mm. (B) Paraffin-embedded cross-sectional images of C3H10T1/2, NIH3T3, and hiPS-MPC sheets on day 5. Scale bars, 50 μm. (C) Representative fluorescence microscopy images of CFSE-stained cell sheets on day 5 used to assess cell viability. CFSE-positive cells are shown in green. Scale bars, 5 mm.

Cell sheets generated from hiPS-MPCs were compared with sheets prepared from mouse MSCs (C3H10T1/2) and NIH3T3. hiPS-MPCs readily formed structurally intact, manipulable sheets, comparable to those produced by these cell types (Figure 2A). Similar sheet formation was observed in mouse and human adipose-derived MSCs (mADSCs and hADSCs) (Figure S1A), indicating that hiPS-MPCs possess sheet-forming properties comparable to primary MSCs. Histological analysis of paraffin-embedded samples revealed that all sheet types exhibited continuous planar structures with a uniform thickness of approximately 20 μm (Figures 2B and S1B), demonstrating consistent structural organization across different cell sources. hiPS-MPC sheets were maintained in culture for 5 days and subjected to live-cell staining using carboxyfluorescein succinimidyl ester (CFSE) to evaluate cell viability within the sheets. Fluorescence microscopy revealed strong and homogeneous CFSE signals throughout the sheets (Figures 2C and S1C), indicating that hiPS-MPCs remained viable within the dense sheet structure. Together, these results demonstrate that hiPS-MPCs can efficiently generate structurally stable and viable cell sheets suitable for transplantation.

### Cell sheet formation prolongs *in vivo* cell survival

**Figure 3:**
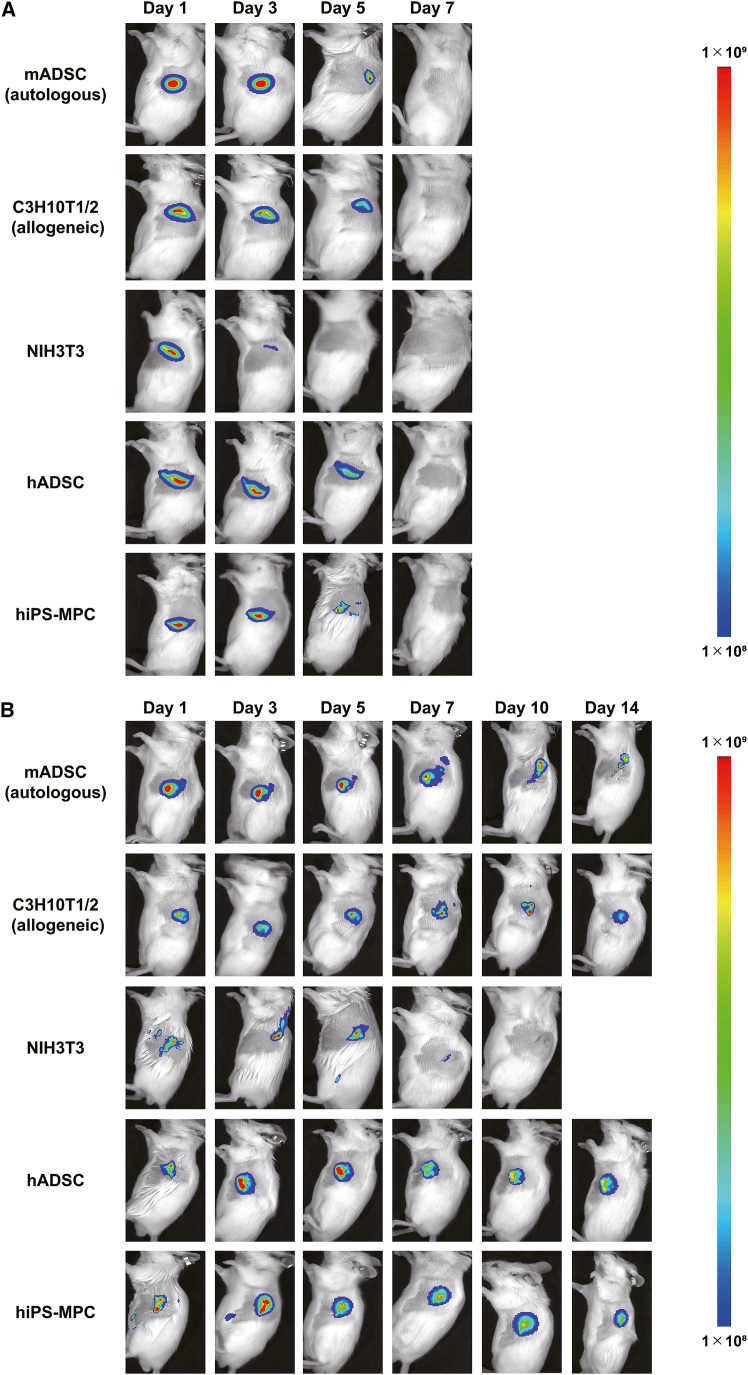
Prolonged *in vivo* survival of transplanted cells by cell sheet formation *In vivo* fluorescence imaging of BALB/c mice following subcutaneous transplantation of DiR-labeled cells. (A) Cell suspension. Cells were transplanted as single-cell suspensions, including mADSCs (autologous), C3H10T1/2 (allogeneic), NIH3T3, hADSCs, and hiPS-MPCs. (B) Cell sheet. The corresponding cells were transplanted as intact cell sheets, including C3H10T1/2, mADSC, NIH3T3, hADSC, and hiPS-MPC sheets. Fluorescence signals represent the persistence of transplanted cells over time.

We compared the *in vivo* persistence of cells transplanted as single-cell suspensions or intact cell sheets to determine whether cell sheet formation enhances post-transplantation cell survival. Cells were labeled with the near-infrared dye 1,1′-dioctadecyl-3,3,3′,3′-tetramethylindotricarbocyanine iodide (DiR) and monitored using longitudinal *in vivo* fluorescence imaging following subcutaneous transplantation into immunocompetent BALB/c mice. When transplanted as single-cell suspensions, signals from all cell types, including mADSCs (primary mouse adipose-derived stromal cells isolated from BALB/c mouse adipose tissue, autologous), C3H10T1/2 (allogeneic), NIH3T3 (allogeneic), hADSCs, and hiPS-MPCs, declined rapidly and became nearly undetectable within 7 days (Figure 3A). In contrast, transplantation of cell sheets markedly improved cell persistence *in vivo*. MSC-derived sheets showed prolonged survival compared with NIH3T3 sheets, which diminished within approximately 10 days of transplantation. Notably, both mADSC and C3H10T1/2 sheets persisted for extended periods under autologous and allogeneic transplantation conditions. Furthermore, hADSC and hiPS-MPC sheets also exhibited prolonged survival following xenotransplantation into immunocompetent mice (Figure 3B). These results demonstrated that cell sheet formation substantially enhances *in vivo* cell persistence compared with cell suspension transplantation. Moreover, MSC-derived sheets, including those generated from hiPS-MPCs, exhibit prolonged survival across autologous, allogeneic, and xenotransplantation settings, whereas NIH3T3 sheets show limited persistence. Based on these results, subsequent experiments focused on C3H10T1/2 for allogeneic transplantation and hiPS-MPCs as representative human MSC-like cells for xenotransplantation.

### Long-term survival and tissue formation following xenotransplantation of hiPS-MPC sheets

**Figure 4:**
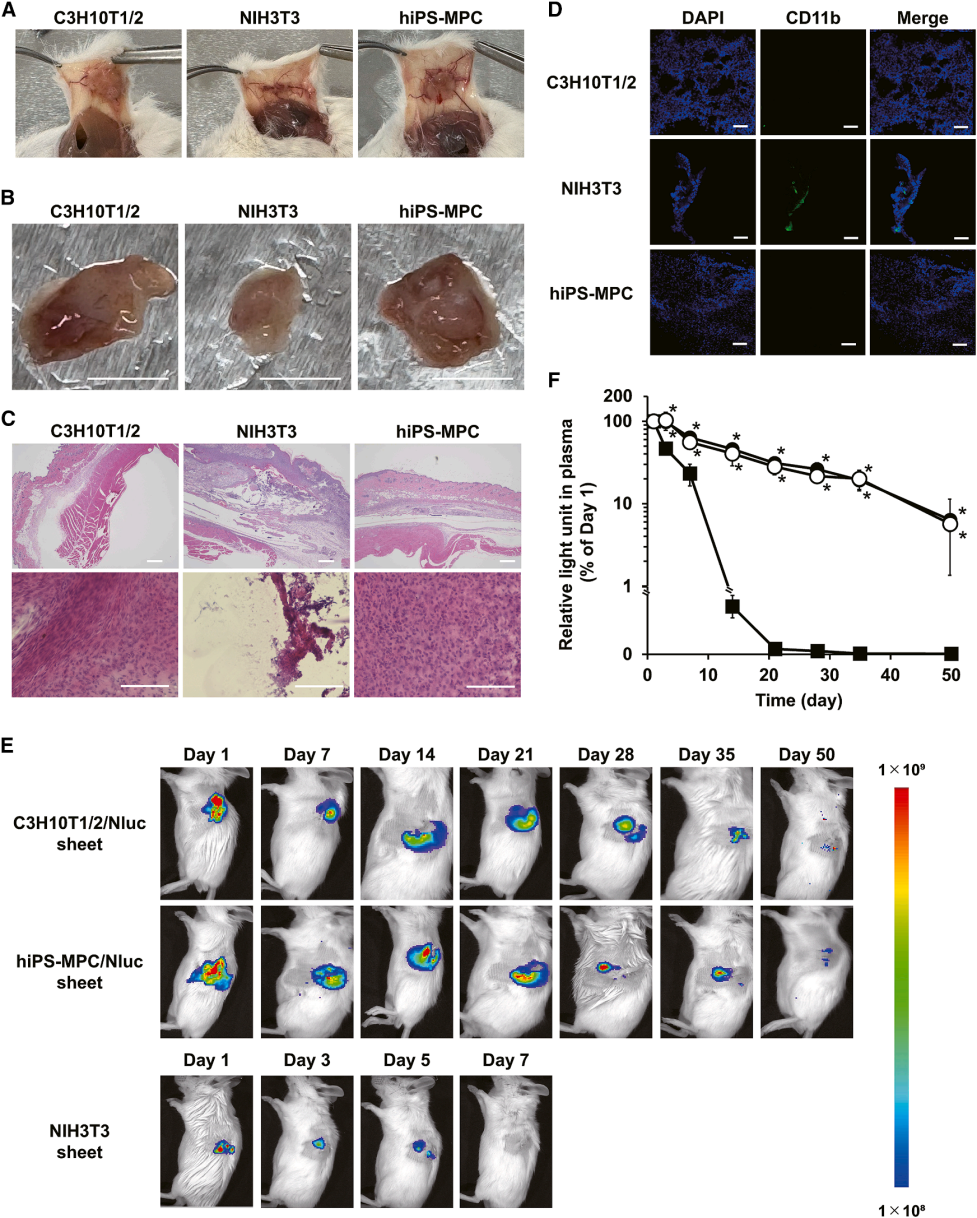
Long-term survival and tissue formation after xenotransplantation of hiPS-MPC sheets (A) Tissue-like structure formation 7 days after transplantation of C3H10T1/2, NIH3T3, and hiPS-MPC sheets into BALB/c mice. (B) Representative macroscopic images of tissue-like structures formed following transplantation of C3H10T1/2, NIH3T3, and hiPS-MPC sheets on day 7. (C) Paraffin-embedded histological sections of skin from BALB/c mice transplanted with C3H10T1/2, NIH3T3, or hiPS-MPC sheets on day 7. Scale bars, 500 μm (upper images) and 200 μm (lower images). (D) Representative immunofluorescence images showing CD11b^+^ macrophage infiltration within tissue-like structures formed after transplantation of C3H10T1/2, NIH3T3, and hiPS-MPC sheets on day 7. Blue, DAPI; green, CD11b. Scale bars, 100 μm. (E) *In vivo* bioluminescence imaging of BALB/c mice following subcutaneous transplantation of NanoLuc (Nluc)-expressing C3H10T1/2, NIH3T3, and hiPS-MPC sheets. Luciferase-derived signals indicate the presence and persistence of viable transplanted cells over time. (F) Quantification of plasma luciferase activity following transplantation of C3H10T1/2/Nluc, NIH3T3/Nluc, and hiPS-MPC/Nluc sheets. Data are presented as the mean ± standard deviation (*n* = 3 mice). Statistical significance was determined using Student’s *t* test. ∗*p* < 0.05 vs. the NIH3T3/Nluc sheet group. White circles, hiPS-MPC/Nluc sheets; black circles, C3H10T1/2/Nluc sheets; black squares, NIH3T3/Nluc sheets.

Cell sheets were transplanted subcutaneously, and the graft sites were analyzed histologically to determine whether hiPS-MPC sheets could survive and form tissue-like structures after xenotransplantation into immunocompetent mice. Both C3H10T1/2 and hiPS-MPC sheets formed well-organized tissue-like structures within the subcutaneous space, which contained abundant blood vessels, indicating active vascularization after transplantation (Figures 4A and 4B). In contrast, the transplantation of NIH3T3 sheets yielded only small, poorly organized structures. Histological examination revealed that the transplanted fibroblast sheets showed a marked structural collapse accompanied by extensive lymphocytic infiltration (Figure 4C). In addition, prominent macrophage accumulation was observed around the grafts, suggesting a strong immune response against the NIH3T3-derived tissues (Figure 4D). In comparison, tissue-like structures generated from C3H10T1/2 and hiPS-MPC sheets maintained their structural integrity and exhibited markedly reduced infiltration of lymphocytes and macrophages (Figures 4C and 4D). These observations suggested that MSC-derived sheets, including hiPS-MPC sheets, can mitigate host immune responses following xenotransplantation. Luciferase-expressing cell sheets were monitored using bioluminescence imaging to evaluate graft survival *in vivo*. Robust luciferase signals derived from C3H10T1/2 and hiPS-MPC sheets persisted for approximately 50 days after transplantation, whereas signals from NIH3T3 sheets declined rapidly and became undetectable within 7 days (Figure 4E). Consistent with the imaging results, plasma luciferase activity confirmed that C3H10T1/2 and hiPS-MPC sheets had significantly prolonged survival compared with NIH3T3 sheets (Figure 4F).

### Tissue-like structure formation after transplantation of hiPS-MPC sheets in nude mice

To further examine whether transplanted cell sheets can survive and form tissue-like structures *in vivo*, C3H10T1/2, hiPS-MPC, and NIH3T3 sheets were transplanted subcutaneously into nude mice. Macroscopic observation of the transplantation sites revealed the formation of tissue-like structures at the sites transplanted with C3H10T1/2 or hiPS-MPC sheets (Figures S2A and S2B). These structures contained visible blood vessels, indicating active vascularization within the graft. In contrast, NIH3T3 sheets failed to generate comparable tissue-like structures. Next, we examined the immunological characteristics of the graft sites. Histological analysis of skin sections after transplantation revealed no apparent inflammatory responses in the C3H10T1/2- or hiPS-MPC sheet-derived tissues, with minimal lymphocyte infiltration (Figure S2C). Consistent with this observation, CD11b-positive macrophage accumulation was detected in these tissues (Figure S2D). In contrast, NIH3T3 sheets showed clear signs of immune rejection, including graft collapse, lymphocyte infiltration, and abundant CD11b-positive macrophages. Analysis of cytokine production within tissue-like structures further supports these findings. C3H10T1/2- and hiPS-MPC sheet-derived tissues exhibited high levels of the anti-inflammatory cytokine TGF-β, whereas NIH3T3-derived tissues showed increased levels of the pro-inflammatory cytokine tumor necrosis factor-α (TNF-α) (Figure S2E). Taken together, these results suggested that MSC-derived sheets, including hiPS-MPC sheets, promote a local anti-inflammatory microenvironment that supports graft survival and tissue formation *in vivo*.

### Xenotransplanted hiPS-MPC sheets promote immune-regulated tissue formation

**Figure 5:**
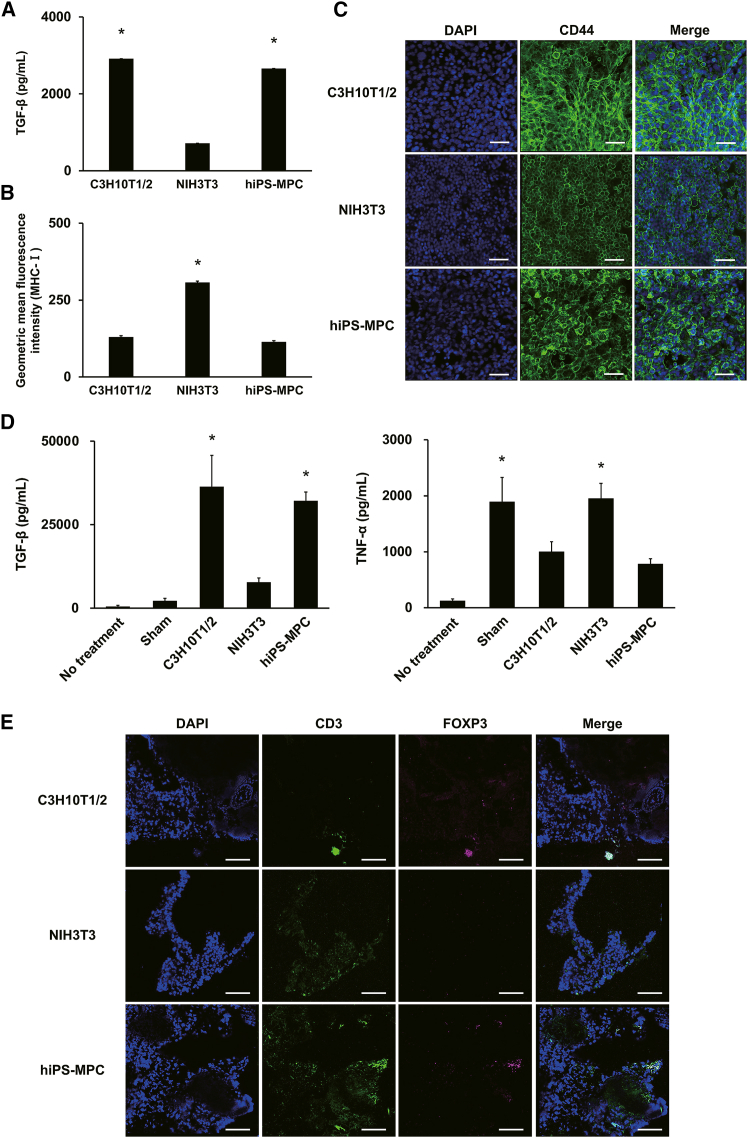
Immune regulatory properties of hiPS-MPC sheets after xenotransplantation (A) Production of anti-inflammatory cytokine TGF-β by C3H10T1/2, NIH3T3, and hiPS-MPC sheets. Data are presented as mean ± standard deviation (*n* = 3 independent experiments). Statistical significance was determined using one-way ANOVA followed by Dunnett’s multiple comparisons test. ∗*p* < 0.05 versus the NIH3T3 group. (B) Flow cytometric analysis of major histocompatibility complex class I (MHC-I) expression in C3H10T1/2, NIH3T3, and hiPS-MPCs. Data are presented as mean ± standard deviation (*n* = 3 independent experiments). Statistical significance was determined using one-way ANOVA followed by Dunnett’s multiple comparisons test. ∗*p* < 0.05 versus the C3H10T1/2 group. (C) Representative immunofluorescence images showing CD44 expression in C3H10T1/2, NIH3T3, and hiPS-MPC sheets. Blue, DAPI; green, CD44. Scale bars, 100 μm. (D) Cytokine production within tissue-like structures formed after transplantation of C3H10T1/2, NIH3T3, and hiPS-MPC sheets into BALB/c mice on day 1. Data are presented as mean ± standard deviation (*n* = 3 mice). Statistical significance was determined using one-way ANOVA followed by Dunnett’s multiple comparisons test. ∗*p* < 0.05 versus the NIH3T3 group (left) or the C3H10T1/2 group (right). (E) Representative immunofluorescence images of CD3^+^ T cells and FOXP3^+^ regulatory T cells within tissue-like structures formed after transplantation of C3H10T1/2, NIH3T3, and hiPS-MPC sheets into BALB/c mice on day 7. Blue, DAPI; green, CD3; magenta, FOXP3. Scale bars, 100 μm.

Because both C3H10T1/2 and hiPS-MPC sheets exhibited prolonged survival after transplantation, we next investigated the mechanisms underlying their immune tolerance. Analysis of culture supernatants revealed abundant secretion of the anti-inflammatory cytokine TGF-β and IL-10 from both C3H10T1/2 and hiPS-MPC sheets (Figures 5A and S3A), suggesting strong immunomodulatory activity. The expression of major histocompatibility complex (MHC) class I and programmed death-ligand 1 (PD-L1) was analyzed using flow cytometry to further evaluate their immunogenic profiles. Both C3H10T1/2 and hiPS-MPCs displayed markedly lower MHC class I expression and higher PD-L1 expression than NIH3T3 (Figures 5B and S3B), indicating a reduced immunogenic phenotype. Immunofluorescence analysis confirmed the strong expression of the MSC marker CD44 in both C3H10T1/2 and hiPS-MPC sheets, which was consistent with their mesenchymal identity (Figures 5C and S3C). Similar findings were obtained using commercially available hiPS-MPCs derived from an independent hiPSC line (Ti003-A), which also exhibited sheet formation, high TGF-β production, and elevated PD-L1 and CD44 expression (Figure S4). These findings suggest that the immunomodulatory and sheet-forming properties of hiPS-MPCs are reproducible across independent hiPSC lines. In addition, MSC-derived sheets exhibited higher levels of the anti-inflammatory cytokine TGF-β and lower levels of the pro-inflammatory cytokine TNF-α than NIH3T3 sheets. (Figure 5D). T cell populations were analyzed using immunofluorescence staining to further characterize the immune microenvironment within the grafts. CD3^+^FOXP3^+^ regulatory T cells were enriched in tissues derived from C3H10T1/2 and hiPS-MPC sheets, suggesting the establishment of an immunosuppressive microenvironment that supports graft tolerance. In contrast, NIH3T3 sheets contained predominantly CD3^+^FOXP3^-^ T cells, consistent with effector T cell activation and immune rejection (Figure 5E). Taken together, these findings indicated that hiPS-MPC sheets promote long-term graft survival and tissue formation through multiple immunomodulatory mechanisms, including the production of anti-inflammatory cytokines, reduced immunogenicity, and induction of regulatory T cell responses.

### Xenogeneic hiPS-MPC sheets form vascularized tissue-like structures and integrate with host tissue *in vivo*

**Figure 6:**
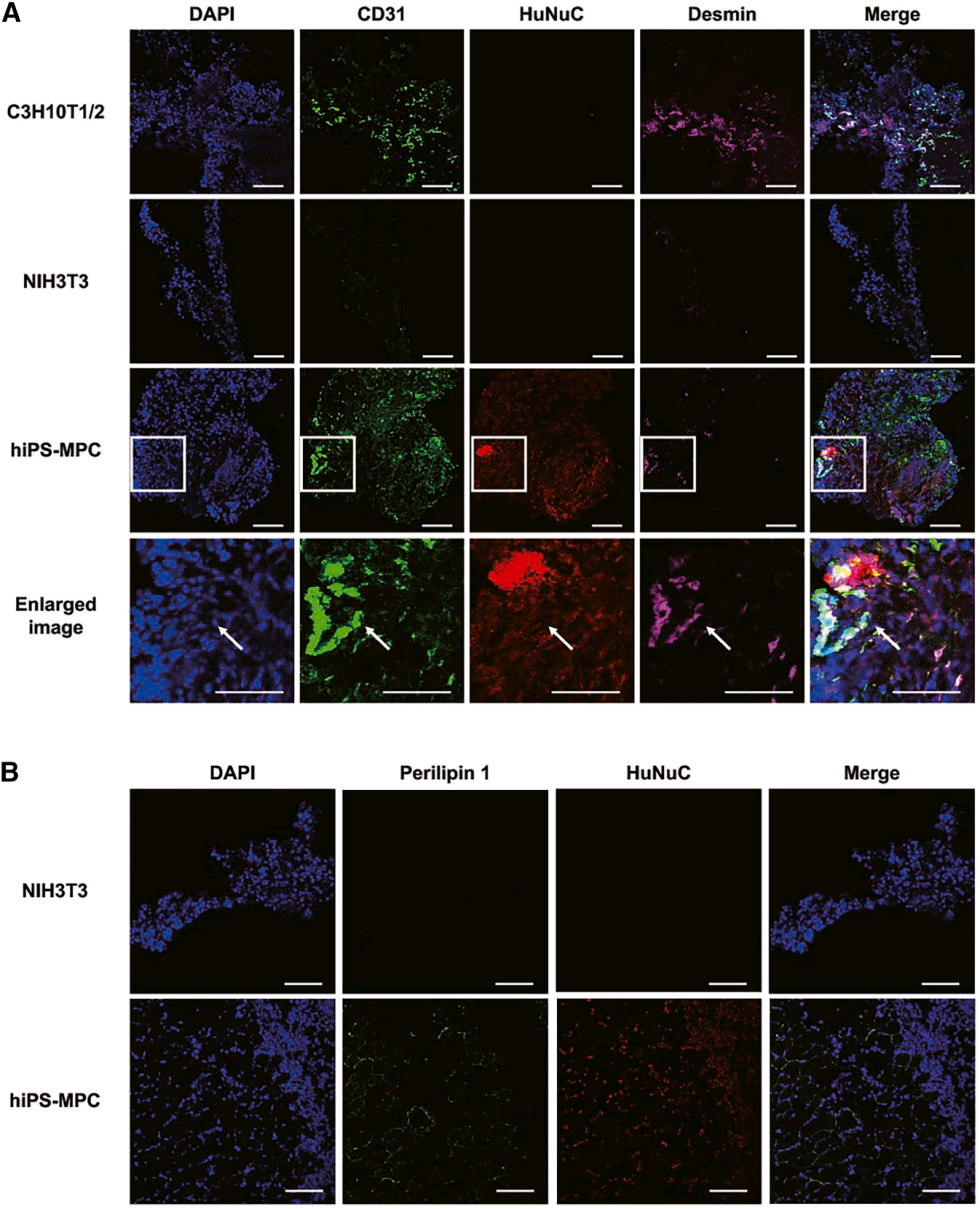
Xenogeneic hiPS-MPC sheets form tissue-like structures and integrate with host tissue *in vivo* (A) Representative immunofluorescence images showing CD31 (endothelial cell marker), human nuclei (HuNuC), and desmin (vascular smooth muscle cell marker) in tissue-like structures formed after transplantation of C3H10T1/2, NIH3T3, and hiPS-MPC sheets into BALB/c mice on day 28. Blue, DAPI; green, CD31; red, HuNuC; magenta, desmin. Scale bars, 100 μm. Arrows, luminal structure. A higher-magnification inset of the boxed region is shown to highlight luminal structures. (B) Representative immunofluorescence images showing perilipin-1 (mature adipocyte marker) and HuNuC in tissue-like structures formed after transplantation of NIH3T3 and hiPS-MPC sheets into BALB/c mice on day 28. Blue, DAPI; green, perilipin-1; red, HuNuC. Scale bars, 100 μm.

Histological analyses revealed the formation of well-organized tissue-like structures following the xenotransplantation of hiPS-MPC sheets. CD31^+^ endothelial cells formed extensive vascular networks within the grafts and were closely associated with Desmin^+^ vascular smooth muscle cells, indicating the development of structurally mature vessels (Figure 6A). These vascular structures frequently exhibited lumen-containing morphologies, suggesting the formation of perfusable vascular networks. Quantitative analysis further demonstrated a significant increase in CD31-positive area and fluorescence intensity in hiPS-MPC-derived tissues compared with control groups, supporting enhanced vascularization (Figure S5). In addition to vascular structures, Perilipin-1^+^ adipocyte-like cells were detected within the grafted tissues, consistent with adipogenic differentiation and the establishment of differentiated stromal compartments (Figure 6B). Although complete lineage tracing was not performed, the presence of HuNuC^+^ nuclei in the grafted tissues suggests that transplanted hiPS-MPC-derived cells contributed, at least in part, to tissue formation. Comparative analysis using UE7T-13 cells, a human bone marrow-derived MSC line, revealed preferential formation of Desmin^+^ and CD31^+^ vascular-associated structures, whereas adipogenic differentiation was not observed under the same conditions (Figure S6). Together, these findings suggest that hiPS-MPC sheets not only survive long after xenotransplantation but also participate in tissue morphogenesis, generating organized, vascularized tissue-like structures *in vivo*.

### hiPS-MPC-based cell sheet platforms promote functional lymphatic recovery in a mouse model of lymphedema

**Figure 7:**
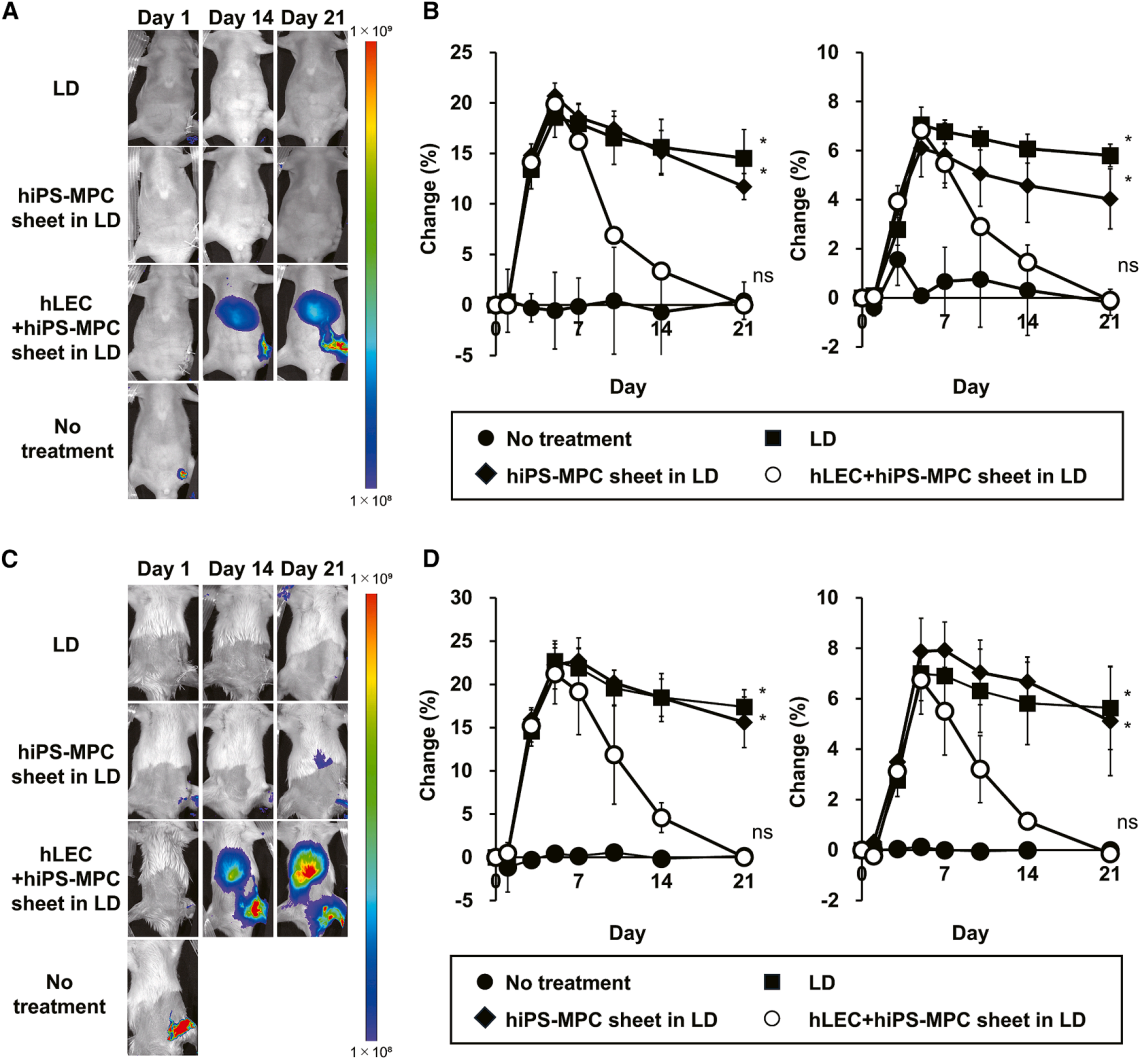
hiPS-MPC-based cell sheet platforms promote functional lymphatic recovery in a mouse model of lymphedema (A) *In vivo* fluorescence imaging after the injection of ICG to footpads of normal (no treatment) or LD mice (BALB/c nude mouse) after cell transplantation. LD mice had their inguinal and popliteal lymph nodes removed. Bioengineered tissues were transplanted to the site of the removed popliteal lymph nodes. (B) Change in the size of the paws (left) and legs (right) of bioengineered tissue-transplanted LD mice (BALB/c nude mouse). The thicknesses of the paws and legs were measured and the change rates from day 0 were calculated. Data are presented as mean ± standard deviation (*n* = 3 mice). Statistical significance was determined using one-way ANOVA followed by Dunnett’s multiple comparisons test. ∗*p* < 0.05 versus the no treatment group. ns, not significant. (C) *In vivo* fluorescence imaging after the injection of ICG to footpads of normal (no treatment) or LD mice (BALB/c mouse) after cell transplantation. LD mice had their inguinal and popliteal lymph nodes removed. Bioengineered tissues were transplanted to the site of the removed popliteal lymph nodes. (D) Change in the size of the paws (left) and legs (right) of bioengineered tissue-transplanted LD mice (BALB/c mouse). The thicknesses of the paws and legs were measured and the change rates from day 0 were calculated. Data are presented as mean ± standard deviation (*n* = 3 mice). Statistical significance was determined using one-way ANOVA followed by Dunnett’s multiple comparisons test. ∗*p* < 0.05 versus the no-treatment group. ns, not significant.

To further evaluate the functional relevance of the hiPS-MPC-based cell sheet platform, we assessed its therapeutic potential in a mouse model of lymphedema. Based on our previous findings that stacked-cell sheets composed of MSCs and lymphatic endothelial cells (LECs) can restore lymphatic function,^12^ we examined whether hiPS-MPC-based sheets could achieve similar therapeutic effects. Following lymphadenectomy (LD), mice developed impaired lymphatic drainage and progressive edema formation. We first evaluated therapeutic effects in nude mice to assess functional outcomes in an immunodeficient setting. Transplantation of hiPS-MPC sheets partially improved lymphatic function, whereas composite sheets consisting of hiPS-MPCs and human LECs (hLECs) resulted in more pronounced therapeutic effects. *In vivo* imaging using indocyanine green (ICG) demonstrated enhanced lymphatic drainage in treated mice, particularly in the hLEC+hiPS-MPC sheet group, compared with untreated controls. Consistent with these findings, quantitative analysis revealed a significant reduction in edema size in treated groups in nude mice (Figures 7A and 7B). We next examined whether similar therapeutic effects could be observed in immunocompetent BALB/c mice. Notably, transplantation of hLEC+hiPS-MPC sheets also improved lymphatic drainage and suppressed edema formation in this setting (Figures 7C and 7D). These findings indicate that hiPS-MPC-based cell sheet platforms can promote functional lymphatic recovery *in vivo* and that their therapeutic effects are maintained across different immune conditions, particularly when combined with lineage-specific endothelial components.

## Discussion

Recently, increasing attention has focused on tissue regeneration using cell sheet transplantation, as this technology preserves cell-cell interactions and extracellular matrix components. This creates a microenvironment that supports cell engraftment and functional integration.^16^^,^^17^ For example, cardiomyocyte cell sheets have been explored as a therapeutic strategy for severe heart disease, where they promote myocardial repair, in part through cytokine-mediated angiogenesis, and clinical trials using hiPSC-derived cardiomyocytes are currently underway.^18^^,^^19^ Although stem cell-derived cell sheets are expected to enhance tissue repair through paracrine signaling and structural support, relatively few studies have demonstrated their capacity to engraft, differentiate, and contribute directly to tissue formation *in vivo.*^20^ In this study, we demonstrated that hiPS-MPC sheets can successfully engraft after xenotransplantation and form organized tissue-like structures *in vivo*. These grafts were characterized by high cellularity and well-developed vascular networks (Figures 4A, 4B, 6A, and 6B). Histological analyses further revealed the presence of differentiated tissue components, including CD31^+^ endothelial cells, Desmin^+^ vascular smooth muscle-like cells, and Perilipin-1^+^ adipocytes (Figure 6B). This indicated the establishment of structurally organized stromal compartments within the grafts. Importantly, no evidence of teratoma formation was observed during the observation period, suggesting that the hiPS-MPCs underwent controlled differentiation and maintained stable engraftment. These properties suggested that hiPS-MPC sheets represent a broadly applicable platform for regenerative tissue construction.

Interaction with host immune cells is a critical determinant of graft survival following transplantation.^21^ MSCs are well known to exert immunosuppressive effects by secreting anti-inflammatory mediators and modulating immune cell activity.^22^ Consistent with these properties, both C3H10T1/2 and hiPS-MPC sheets produced high levels of the anti-inflammatory cytokine TGF-β and IL-10 prior to transplantation, and tissue-like structures derived from these sheets maintained sustained anti-inflammatory cytokine levels *in vivo* (Figures 5A–5D, S2E, and S3A). In contrast, NIH3T3 sheets elicited acute inflammatory responses, did not form stable tissue-like structures, and were rapidly rejected after transplantation (Figures 4C and 4D), suggesting a potential role for the immunomodulatory capacity of stromal cells in graft engraftment. Longitudinal *in vivo* tracking further demonstrated that C3H10T1/2 and hiPS-MPC sheets survived for at least 50 days in immunocompetent mice, whereas NIH3T3 sheets were lost within one week (Figures 4E and 4F). The prolonged persistence of the MSC-derived sheets likely reflects multiple complementary immunomodulatory mechanisms. These include cytokine-mediated suppression of inflammatory responses, recruitment of CD3^+^FOXP3^+^ regulatory T cells within the graft microenvironment (Figure 5E), and intrinsically low immunogenicity characterized by reduced MHC class I expression and elevated PD-L1 expression (Figures 5B and S3B). Taken together, these findings indicated that MSC-derived cell sheets establish a local immunoregulatory microenvironment that promotes graft tolerance and enables durable engraftment in immunocompetent hosts.

Other mechanisms may also contribute to the stable engraftment and long-term persistence of the hiPS-MPC sheets. CD44, a cell surface glycoprotein involved in cell adhesion and homing, enhances the engraftment and survival of transplanted MSCs.^23^^,^^24^ Consistent with these reports, both C3H10T1/2 and hiPS-MPC sheets expressed high levels of CD44 (Figures 5C and S3B), which likely facilitated stable attachment to the host tissue and may have acted synergistically with immunomodulatory mechanisms to support long-term graft survival. Angiogenesis represents another critical factor for graft viability, as vascular integration ensures adequate nutrient delivery and waste removal.^25^ MSCs secrete angiogenic factors such as vascular endothelial growth factor and fibroblast growth factor that promote vascular remodeling and tissue integration via paracrine signaling.^26^ The findings of this study suggested that transplanted cells may also play a more direct role in vascular maturation in addition to these paracrine effects. In agreement with these properties, C3H10T1/2 and hiPS-MPC sheets generated abundant and well-organized vasculature, including mature CD31^+^/Desmin^+^ vessel structures (Figures 4A, 4B, and 6A), with HuNuC^+^ human cells directly contributing to vascular smooth muscle-like compartments. These findings indicated that the transplanted cells actively participate in vascular maturation rather than acting solely through paracrine signaling. Furthermore, hiPS-MPCs retained multilineage differentiation potential *in vivo* and formed Perilipin-1^+^ adipocytes within the grafts (Figure 6B), demonstrating physiological lineage commitment without evidence of dysplastic changes. In contrast, comparative analysis using UE7T-13 cells revealed a distinct tissue-forming profile characterized by the formation of Desmin^+^ and CD31^+^ vascular-associated structures, with no detectable adipogenic differentiation under the same conditions (Figure S6). These differences suggest that hiPS-MPCs exhibit a functional phenotype more closely resembling adipose-derived MSCs, whereas bone marrow-derived MSCs may preferentially contribute to vascular and stromal support. Taken together, these findings indicate that hiPS-MPCs can adapt to the host microenvironment and may contribute to the formation of stromal and vascular tissues.

In addition to promoting tissue organization and vascularization, our results demonstrate that hiPS-MPC-based cell sheet platforms can contribute to functional tissue repair *in vivo*. Using an LD mouse model, we observed improved lymphatic drainage and reduced edema formation following transplantation of hiPS-MPC-based sheets (Figure 7). Notably, these therapeutic effects were observed in both immunodeficient and immunocompetent settings (Figures 7A–7D), indicating that the functional benefits of this platform are maintained across different immune environments. Furthermore, the enhanced efficacy observed with composite sheets containing LECs suggests that the regenerative potential of hiPS-MPC-based platforms can be augmented through combination with lineage-specific cell types. While this composite system does not isolate the effects of hiPS-MPCs alone, these findings support the concept that hiPS-MPC-based cell sheet platforms provide a versatile foundation for functional regenerative therapies.

Collectively, these findings highlight the versatility of hiPS-MPC sheets as a regenerative platform. By simultaneously promoting vascularization, stromal diversification, and immunomodulation, hiPS-MPC sheets generate organized, functional tissue-like structures across autologous, allogeneic, and xenogeneic transplantation settings. Their ability to survive long-term, differentiate into multiple cell types, and integrate with the host tissue underscores their potential as a broadly applicable strategy for tissue regeneration. These characteristics suggest that hiPS-MPC sheets may serve as a versatile cell-based platform for repairing or reconstructing diverse tissues and provide a promising foundation for future translational applications in regenerative medicine.

### Limitations of the study

This study has several limitations. First, although the xenotransplantation model in immunocompetent mice provides a stringent platform for evaluating graft persistence and host immune responses, the responses observed in this setting may not fully reflect those in clinical allogeneic or autologous transplantation. Second, although our findings suggest that TGF-β and IL-10 production, reduced MHC-I expression, increased PD-L1 and CD44 expression, and regulatory T cell recruitment contribute to the prolonged survival of hiPS-MPC sheets, the relative contributions of these mechanisms remain to be fully elucidated. Third, complete lineage tracing was not performed, and therefore the direct contribution of transplanted hiPS-MPCs to each differentiated tissue component requires further investigation. Finally, longer-term studies in clinically relevant transplantation models will be required to further evaluate the durability, safety, and therapeutic potential of hiPS-MPC sheet transplantation.

## Resource availability

### Lead contact

Further information and requests for resources and reagents should be directed to and will be fulfilled by the Lead Contact, Dr. Kosuke Kusamori (kusamori@rs.tus.ac.jp).

### Materials availability

This study did not generate new unique reagents or materials.

### Data and code availability

The datasets generated during this study are available from the corresponding author upon reasonable request. This paper does not report original code. Any additional information required to reanalyze the data reported in this paper is available from the lead contact upon reasonable request.

## Acknowledgments

We thank the Biopathology Institute Co., Ltd. for their kind support in some experiments, Professor James M. Wells (10.13039/100007172Cincinnati Children's Hospital Medical Center, Cincinnati, OH, USA) for providing iPS72.3 cells, and Yukiya Takayama (Tokyo University of Science, Tokyo, Japan) for technical guidance in the differentiation of hiPS-MPCs. This work was supported by a Grant-in-Aid for Scientific Research (B) from the 10.13039/501100001691Japan Society for the Promotion of Science (grant nos 23H03749 and 23K28437 to K. K.), a Noguchi Shitagau Research Grant from the 10.13039/501100012017Noguchi Institute (to K. K.), the Top Researcher Development Support Program of Tokyo University of Science (to K. K.), JST 10.13039/501100025019SPRING (grant no. JPMJSP2151 to S. O.), and a Grant-in-Aid for 10.13039/501100001691JSPS Fellows (grant no. 25KJ2110 to S.O.). The graphical abstract was prepared using scientific illustration materials provided by Science Graphics Inc. (Kyoto, Japan). The authors thank Editage for English-language editing of this manuscript.

## Author contributions

S.O. and K.K. conceptualized the study. S.O. and K.K. performed the study investigation. S.O. performed the experiments. S. O., J. M., S. T., S. I., M. N., and K. K. reviewed the data. S.O., S.T., S.I., M.N., and K.K. wrote the manuscript. All authors have read and revised the manuscript.

## Declaration of interests

The authors declare no competing interests.

## Declaration of generative AI and AI-assisted technologies in the writing process

We also used ChatGPT (OpenAI; accessed October 2025–July 2026) to assist with language editing and manuscript refinement, solely to improve grammar and readability and to draft figure legends, and it was not used to generate scientific content or analyze data.

## STAR★Methods

### Key resources table

**Table:** 

REAGENT or RESOURCE	SOURCE	IDENTIFIER
**Antibodies**		

Rat monoclonal anti-CD44	Thermo Fisher Scientific	Cat#16-0441-82; RRID: AB_468955
Rat monoclonal anti-MHC Class I	Thermo Fisher Scientific	Cat# MA5-51813; RRID: AB_3250505
Rabbit monoclonal anti-PD-L1	Cell Signaling Technology	Cat#60475; RRID: AB_2924680
Rat monoclonal anti-CD11b	Abcam	Cat#ab8878; RRID: AB_306831
Rat monoclonal anti-CD3 epsilon	Arigo	Cat# ARG22819; RRID: N/A
Goat polyclonal anti-FOXP3	Thermo Fisher Scientific	Cat# PA5-142112; RRID: AB_2932773
Rat monoclonal anti-CD31	Bio-Techne	Cat#NB600-1475; RRID: AB_789108
Rabbit polyclonal anti-Desmin	Thermo Fisher Scientific	Cat# MA5-38096; RRID: AB_2898014
Rabbit monoclonal anti- Perilipin-1	Cell Signaling Technology	Cat#9349; RRID: AB_10829911
Mouse monoclonal anti-Nuclei Antibody, clone 235-1, Cy3 conjugate	Sigma-Aldrich	Cat# MAB1281C3; RRID: N/A
Goat polyclonal anti-Rat IgG Alexa 488	Thermo Fisher Scientific	Cat# A-11006; RRID: AB_2534074
Donkey polyclonal anti-Rabbit IgG Alexa 488	Thermo Fisher Scientific	Cat# A-21206; RRID: AB_2535792
Donkey polyclonal anti-Rabbit IgG Alexa 647	Thermo Fisher Scientific	Cat# A-31573; RRID: AB_2536183
Human Mesenchymal Stem Cell Marker Antibody Panel	Bio-Techne	Cat# SC017

**Biological samples**		

Human primary adipose-derived mesenchymal stromal cells	Lonza	Cat# PT-5006
Human primary lymphatic endothelial cells	Takara Bio	Cat# C-12216

**Critical commercial assays**		

STEMdiff Mesenchymal Progenitor kit	STEMCELL Technologies	Cat#ST-05240
Mesenchymal Stem Cell Adipogenic Differentiation Medium 2	PromoCell	Cat# C-28016
Mesenchymal Stem Cell Osteogenic Differentiation Medium	PromoCell	Cat# C-28013
Mesenchymal Stem Cell Chondrogenic Differentiation Medium	PromoCell	Cat# C-28012
Nano-Glo Luciferase Assay System	Promega	Cat# N1110
ELISA MAX Deluxe Set Mouse TNF-α	BioLegend	Cat#430904
ELISA MAX Deluxe Set Mouse TGF-β	BioLegend	Cat#433007
ELISA MAX Deluxe Set Human TGF-β	BioLegend	Cat#432907
ELISA MAX Deluxe Set Mouse IL-10	BioLegend	Cat#431414

**Deposited data**		

Raw and analyzed data	This paper	Source Data file

**Experimental models: Cell lines**		

Mouse mesenchymal stromal cell line C3H10T1/2, Clone8	ATCC	C3H10T1/2, Clone8
Mouse fibroblast cell line NIH3T3	RIKEN BRC	NIH3T3
Human mesenchymal stromal cell line UE7T-13	Japanese Collection of Research Bioresources	UE7T-13
hiPSC 72.3	Professor James M. Wells (Cincinnati, OH, USA)	hiPSC 72.3
hiPS-MPC derived from the hiPSC line Ti003-A	STEMCELL Technologies	hiPS-MPC derived from the hiPSC line Ti003-A

**Experimental models: Organisms/strains**		

Mouse: BALB/c-nu/nu	Sankyo Labo Service Co	https://www.sankyolabo.co.jp
Mouse: BALB/c	Sankyo Labo Service Co	https://www.sankyolabo.co.jp

**Software and algorithms**		

ImageJ	National Institutes of Health	https://imagej.nih.gov/ij/
FlowJo software version 10.7.2	BD Biosciences Pharmingen	https://flowjo.com/previous-versions-flowjo
JMP Student Edition 18	SAS Institute Inc.	https://www.jmp.com

### Experimental model and study participant details

#### Experimental animals

Male BALB/c and BALB/c-nu/nu mice (5–8 weeks old) were purchased from Sankyo Labo Service Co., Inc. (Tokyo, Japan) and maintained under specific pathogen-free conditions with free access to food and water. All animal experiments were approved by the Institutional Animal Experimentation Committee of Tokyo University of Science (latest approval number: Y23004; latest approval date: August 8, 2024) and performed in accordance with the National Institutes of Health Guide for the Care and Use of Laboratory Animals and ARRIVE guidelines. Only male mice were used throughout the study to minimize biological variability. Sex was therefore not evaluated as an experimental variable.

#### Cell lines

Mouse mesenchymal stromal cell line C3H10T1/2, Clone8 was obtained from the American Type Culture Collection (ATCC) (Manassas, VA, USA). The mouse fibroblast cell line NIH3T3 was obtained from RIKEN Bioresource Center (RIKEN BRC) (Ibaraki, Japan). Human mesenchymal stromal cell line UE7T-13 was obtained from Japanese Collection of Research Bioresources Cell Bank (Osaka, Japan). Human iPSCs (hiPSC 72.3) were kindly provided by Professor James M. Wells (Cincinnati, OH, USA). The donor age and sex of hiPSC 72.3 were not available because the cell line was de-identified. hiPS-MPC derived from the hiPSC line Ti003-A, which was established from a 48 year old female donor, were purchased from STEMCELL Technologies (Vancouver, Canada).

#### Primary cells

Mouse primary adipose-derived mesenchymal stromal cells (mADSCs) were isolated from the adipose tissue of male BALB/c mice (5–7-week-old). Human primary adipose-derived mesenchymal stromal cells (hADSCs), isolated from the adipose tissue of a 37 year old female donor, were purchased from Lonza (Basel, Switzerland, PT-5006, lot: 21TL347548). Human primary lymphatic endothelial cells (hLEC) were purchased from Takara Bio Inc. (Tokyo, Japan, C-12216, lot: 467Z001.2). The hLECs were isolated from juvenile foreskin of a 2 year old male donor. Commercially obtained human primary cells were isolated from donors who had provided informed consent under protocols approved by the supplier’s institutional ethics committee.

#### Authentication

Commercially obtained cell lines were authenticated by the suppliers prior to distribution. No additional authentication was performed by the authors. All cell lines used in this study were routinely tested for mycoplasma contamination using a PCR-based detection kit and confirmed to be mycoplasma-free.

### Method details

#### Cell culture

C3H10T1/2, NanoLuc luciferase (Nluc)-expressing C3H10T1/2 (C3H10T1/2/Nluc), mADSCs, and UE7T-13 were cultured in DMEM supplemented with 15% heat-inactivated FBS and 1% penicillin-streptomycin-L-glutamine solution. NIH3T3 and Nluc gene-expressing NIH3T3 (NIH3T3/Nluc) were cultured in DMEM supplemented with 10% heat-inactivated FBS and 1% penicillin-streptomycin-L-glutamine. hiPSCs were cultured in mTeSR Plus Medium (STEMCELL Technologies). hiPS-MPCs, Nluc gene-expressing hiPS-MPCs (hiPS-MPC/Nluc), hADSCs, and hiPS-MPCs (Ti003-A) were cultured in Mesenchymal Stem Cell Growth Medium 2 (PromoCell, Heidelberg, Germany). hLECs were cultured in Endothelial Cell Media MV2 (Takara Bio Inc.) and maintained in a humidified atmosphere containing 5% CO_2_ at 37°C.

#### Generation and characterization of hiPS-MPCs

MPCs were differentiated using a STEMdiff Mesenchymal Progenitor kit (STEMCELL Technologies). Briefly, iPSCs were first induced to differentiate into early mesodermal progenitor cells in STEMdiff-ACF Mesenchymal Induction Medium (STEMCELL Technologies) for 4 days, and the medium was changed to MesenCult-ACF Plus Medium (STEMCELL Technologies) for another 2 days. Cells were passaged and cultured in MesenCult-ACF Plus Medium (STEMCELL Technologies) in an Animal Component-Free Cell Attachment Sub (STEMCELL Technologies)-coated 100 mm dish. hiPS-MPCs were differentiated into adipocytes, osteoblasts, and chondrocytes using Mesenchymal Stem Cell Adipogenic Differentiation Medium 2, Mesenchymal Stem Cell Osteogenic Differentiation Medium, and Mesenchymal Stem Cell Chondrogenic Differentiation Medium (PromoCell). After differentiation, the cells were stained with Oil Red O, Alizarin Red S, and Alcian blue, as reported previously.^27^ Furthermore, MSCs surface markers of hiPS-MPCs were characterized using fluorescent antibodies and flow cytometry. The antibodies used were obtained from the Human Mesenchymal Stem Cell Marker Antibody Panel (Bio-Techne, Minneapolis, MN, USA, SC017).

#### Fabrication of cell sheets

Cell sheets were prepared as previously described.^12^ Briefly, C3H10T1/2, NIH3T3, or hiPS-MPCs (1×10^6^ cells) were seeded into 24-well transwell inserts (Corning, NY, USA) and placed into a culture plate. The culture plate containing the seeded cells was immediately centrifuged at 750 × *g* for 30 s to allow the cells to settle at the bottom of the inserts. Cell sheets were cultured for 24 h with a medium change to allow sheet formation. To prepare a stacked-cell sheet, hiPS-MPCs (7.5×10^5^ cells) were seeded into 24-well Transwell inserts (Corning) and set into a culture plate. The culture plate with seeded cells was immediately centrifuged at 750 ×g and 30 s. After 24 h-incubation, hLEC (2×10^5^ cells) were gently seeded on the layered hiPS-MPC sheet. After another 24 h-incubation, hiPS-MPC (7.5×10^5^ cells) were gently seeded on hLECs and hiPS-MPCs in the inserts using the same procedure. Then, the cells were cultured for 5 days with changes of medium.

#### Characterization of the cell sheets

The cells were labeled with carboxyfluorescein succinimidyl ester (CFSE) prior to sheet formation to assess the cell survival and spatial distribution within the fabricated cell sheets. Briefly, C3H10T1/2, NIH3T3, or hiPS-MPCs were incubated with 10 μM CFSE for 30 min at 37 °C, washed thoroughly with culture medium to remove excess dye, and subsequently seeded (1×10^6^ cells) into 24-well Transwell inserts as described above. Culture plates were immediately centrifuged at 750 × *g* for 30 s to facilitate cell aggregation at the bottom of the inserts. The cells were then cultured for 24 h to allow sheet formation. After sheet fabrication, CFSE fluorescence was observed using a BZ-X800 fluorescence microscope (Keyence, Osaka, Japan) to evaluate the cell survival and distribution within the sheets. The culture supernatants were collected after 24 h of incubation to evaluate the cytokine secretion profile of the fabricated cell sheets. The concentrations of TGF-β and IL-10 in the supernatants were quantified using ELISA MAX Deluxe Sets appropriate for each target cytokine (BioLegend, San Diego, CA, USA) according to the manufacturer’s instructions.

#### Histology and immunofluorescence

Cell sheets prepared in Transwell inserts were fixed with 4% paraformaldehyde (PFA), embedded in paraffin, and sectioned at 5 μm thickness. Paraffin sections were prepared by Biopathology Institute Co. Ltd. (Oita, Japan). Paraffin sections were subjected to hematoxylin and eosin (H&E) staining to evaluate the overall morphology and cellular organization. Stained sections were observed under a BZ-X800 fluorescence microscope (Keyence). The transplanted cell sheets containing the Transwell membrane and skin tissue were excised using scissors, embedded in O.C.T. compound (Sakura Finetek, Torrance, CA, USA), and frozen using liquid nitrogen. The frozen tissues were stored at −80 °C until sectioning. The frozen sections were cut to a thickness of 7 μm using a CM3050 S cryostat (Leica Biosystems, Wetzler, Germany). Cell sheets and cryosectioned tissues were fixed with 4% PFA, permeabilized with methanol-dimethyl sulfoxide-hydrogen peroxide mixture (6:1:1), and blocked with 1% bovine serum albumin for immunofluorescence staining. Cell sheets and cryosectioned tissues were stained with primary antibodies and the corresponding secondary antibodies. Nuclei were stained with Vectashield Antifade Mounting Medium with DAPI (Vector Laboratories Inc., Burlingame, CA, USA). Immunofluorescence images were obtained using a Leica SP8 laser scanning confocal microscope with the LAS X Life Science software (Leica Microsystems). The primary and secondary antibodies were as follows: 1:500 CD44 (Thermo Fisher Scientific, Inc., Waltham, MA, USA, 16-0441-82), 1:500 MHC Class I (Thermo Fisher Scientific, MA5-51813), 1:500 PD-L1 (Cell Signaling Technology, Inc., Danvers, MA, USA, 60475), 1:50 CD11b (Abcam, Cambridge, UK, ab8878), 1:100 CD3 (Arigo, Zhubei, Taiwan, ARG22819), 1:200 FOXP3 (Thermo Fisher Scientific, PA5-142112), 1:500 CD31 (Bio-Techne, NB600-1475), 1:100 Perilipin-1 (Cell Signaling Technology, Inc., 9349), 1:100 Desmin (Thermo Fisher Scientific, MA5-38096), 1:50 HuNuC (Sigma-Aldrich, St. Louis, MO, USA, MAB1281C3), 1:500 anti-rat Alexa 488 (Thermo Fisher Scientific, A-11006), 1:500 anti-rabbit Alexa 488 (Thermo Fisher Scientific, A21206) 1:500 anti-rabbit Alexa 647 (Thermo Fisher Scientific, A31573).

#### Subcutaneous transplantation and *in vivo* imaging

Cell sheets containing Nluc-expressing cells or 1 μg/mL 1,1′-dioctadecyl-3,3,3′,3′-tetramethylindotricarbocyanine iodide (DiR, Thermofisher Scientific)-labeled cells were subcutaneously transplanted to BALB/c mice. To detect cell luminescence, 5 mg Nano-Glo Luciferase Assay System (Promega) dissolved in 100 μL PBS was injected into the transplantation site of mice, and luminescence was detected using an IVIS Lumina III (Revvity, Waltham, MA, USA). Blood of mice after transplantation of cell sheets containing Nluc-expressing cells was collected daily from the posterior facial vein, and luciferase activity in the blood was measured using a 2104 EnVision multilabel plate reader (PerkinElmer) for the quantitative evaluation of cell survival. Cell fluorescence was detected using an IVIS Lumina III (Revvity). The cell sheets were then subcutaneously transplanted into BALB/c mice. The tissue-like structures were excised and homogenized 1 or 7 days after transplantation. Subsequently, the TGF-β and TNF-α levels in the homogenates was quantified using mouse TGF-β and TNF-α ELISA MAX Deluxe Sets (BioLegend). Cytokine levels were normalized to the tissue weight.

#### Flow cytometry analysis

The surface expression of MHC class I, PD-L1, and CD44 was analyzed using flow cytometry. Data acquisition was performed on a flow cytometer using a BD FACSLyric (BD Biosciences Pharmingen, San Diego, CA, USA), and at least 10,000 live events were collected per sample. Compensation was performed using the single-stained controls. The data were analyzed using FlowJo software version 10.7.2 (BD Biosciences Pharmingen). MHC class I expression and elevated PD-L1 and CD44 levels were quantified as the mean fluorescence intensity (MFI) and percentage of positive cells.

#### Mouse lymphedema model and evaluation of lymphatic function

LD mice were induced by removing the popliteal and inguinal lymph nodes in the right lower limbs of mice as previously described.^12^ Briefly, skin near the inguinal region of the right lower limb of mice was cut and the popliteal and inguinal lymph nodes were removed. hiPS-MPC sheet or hLEC+hiPS-MPC sheet were transplanted to the site of the removed popliteal lymph node. Thirty μg indocyanine green (ICG, MP Biomedicals, Irvine, CA, USA) dissolved in 30 μL PBS was administered into the footpad of the right lower limb of mice 1, 14, and 21 days after transplantation of the cell sheets. Then, fluorescence at the site of the removed popliteal lymph nodes (the transplantation site) was detected 1 h after ICG administration using IVIS Lumina III (Revvity). For quantitative evaluation, the thicknesses of the paws and legs of the right lower limbs of LD mice with or without transplantation of cells or bioengineered tissues were measured and the change rate relative to day 0 was calculated.

#### Image analysis

CD31 immunofluorescence images were analyzed using ImageJ software (National Institutes of Health, Bethesda, MD, USA). All images were acquired under identical exposure and microscope settings. For quantitative analysis, images were converted to 8-bit grayscale and thresholded using a consistent threshold value across all samples. The percentage of CD31-positive area was calculated for each field. Fluorescence intensity of CD31 was also quantified by measuring the mean gray value within the defined regions of interest. Background signals were subtracted using regions without specific staining. At least three independent fields per sample were analyzed, and the average values were used for statistical analysis.

### Quantification and statistical analysis

Statistical differences were evaluated using one-way analysis of variance (ANOVA), followed by Dunnett’s test for multiple comparisons or Student’s *t* test for comparisons between two groups. All statistical analyses were performed using JMP Student Edition 18 (SAS Institute Inc., Cary, NC, USA). Data are presented as mean ± standard deviation (SD) unless otherwise indicated. Unless otherwise indicated, n represents the number of independent biological replicates (independent cell culture experiments or individual animals, as appropriate). The statistical tests used, exact *n* values, and significance levels for each experiment are provided in the corresponding figure legends. Statistical significance was set at *p* < 0.05. Unless otherwise stated in the figure legends, each experiment was independently repeated at least three times with similar results. No statistical method was used to predetermine sample size. No data were excluded from the analyses. The experiments were not randomized. The investigators were not blinded to allocation during experiments and outcome assessment.

## Supplemental information

Document S1. Figures S1–S6

Data S1. Source data underlying the quantitative analyses presented in the main and supplemental figures
