# Supplementary material for: Human iPSC-derived mesenchymal progenitor cell sheets promote tissue formation after xenotransplantation
Source: iScience. 2026 Aug 11;29(8):117128. doi: 10.1016/j.isci.2026.117128 (PMC13486853; doi:10.1016/j.isci.2026.117128)
Supplement: Supplementary file 1 [file mmc1.pdf]

iScience, Volume *29*

Supplemental information

Human iPSC-derived mesenchymal progenitor cell sheets promote tissue formation after xenotransplantation

Shu Obana, Junta Minato, Saki Takahashi, Shoko Itakura, Makiya Nishikawa, and Kosuke Kusamori

Supplementary Figures

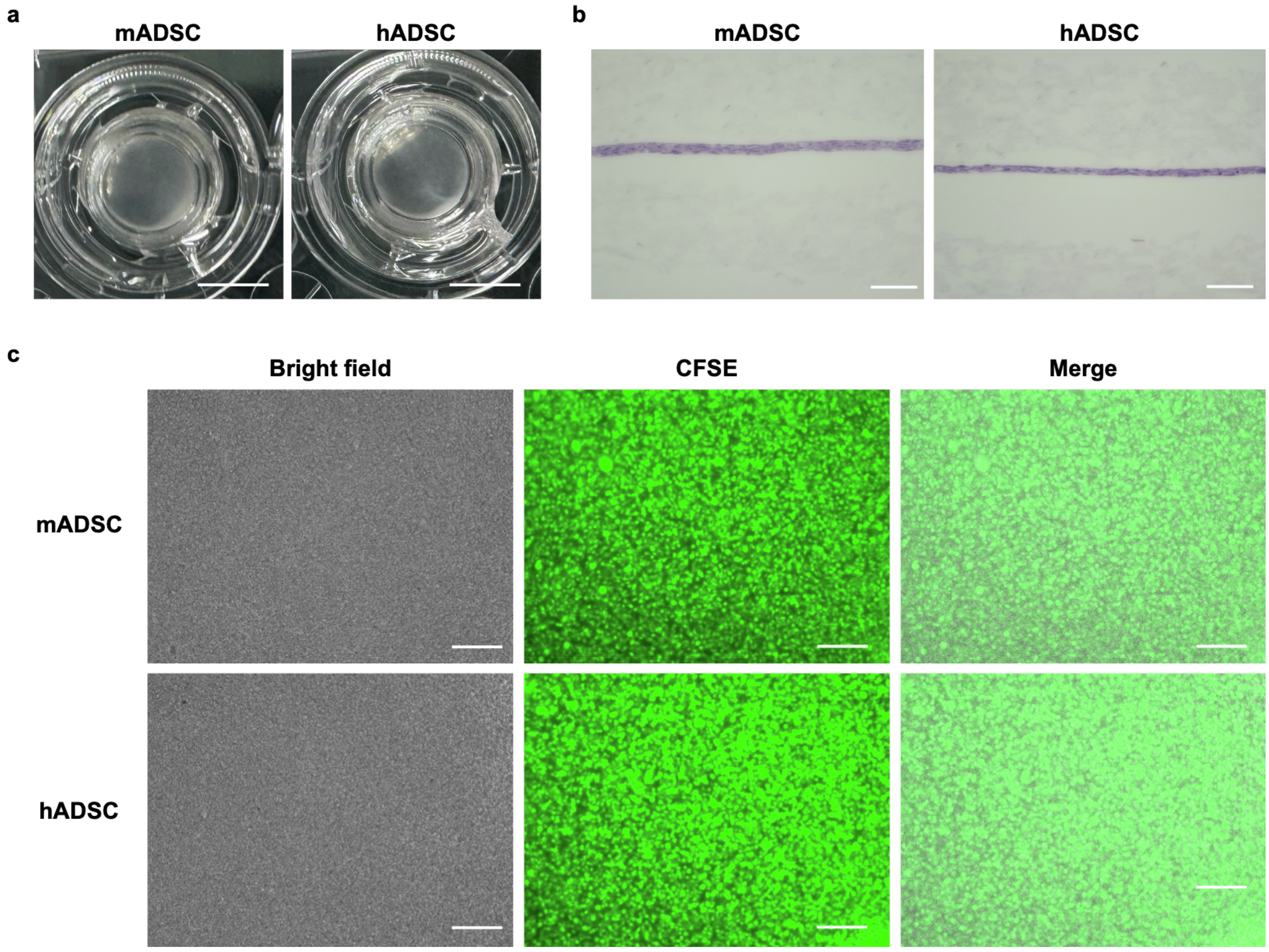

Supplementary Figure S1. Fabrication of mADSC and hADSC sheets.
(a) Representative macroscopic images of murine adipose-derived stromal cell (mADSC) sheets and human adipose-derived stromal cell (hADSC) sheets on day 5. Scale bars, 5 mm. (b) Paraffin-embedded cross-sectional images of mADSC and hADSC sheets on day 5. Scale bars, 50 µm. (c) Representative fluorescence microscopy images of CFSE-stained cell sheets on day 5 for evaluation of cell viability. CFSE-positive cells are shown in green. Scale bars, 500 µm.

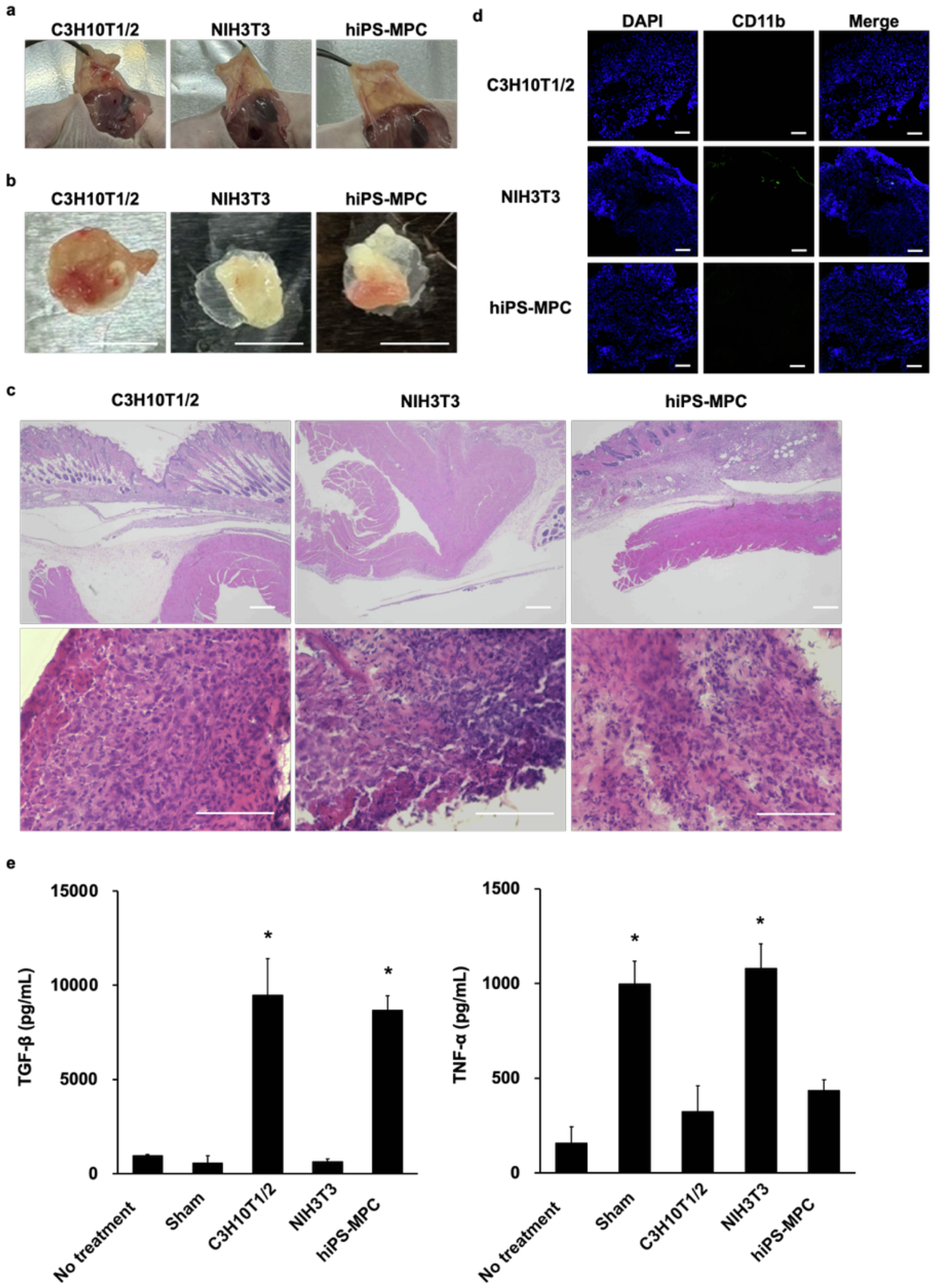

Supplementary Figure S2. Tissue-like structure formation after transplantation of cell sheets into immunodeficient nude mice.

(a) Tissue-like structure formation 7 days after transplantation of C3H10T1/2, NIH3T3, and hiPS-MPC sheets into BALB/c-*nu*/*nu* mice. (b) Representative macroscopic appearance of tissue-like structures formed following transplantation of C3H10T1/2, NIH3T3, and hiPS-MPC sheets on day 7. (c) Paraffin-embedded histological sections of skin from BALB/c-*nu*/*nu* mice transplanted with C3H10T1/2, NIH3T3, and hiPS-MPC sheets on day 7. Scale bars, 500 μm (upper panels) and 200 μm (lower panels). (d) Representative immunofluorescence images showing infiltration of $CD11b^{+}$ macrophages within tissue-like structures formed after transplantation of C3H10T1/2, NIH3T3, and hiPS-MPC sheets on day 7. Blue, DAPI; green, CD11b. Scale bars, 100 μm. (e) Cytokine production within tissue-like structures formed after transplantation of C3H10T1/2, NIH3T3, and hiPS-MPC sheets into BALB/c-nu/nu mice on day 1. Data are presented as mean ± standard deviation (n = 3 mice). Statistical significance was determined using one-way ANOVA followed by Dunnett's multiple comparisons test. *$P < 0.05$ versus the NIH3T3 group (left) or the C3H10T1/2 group (right).

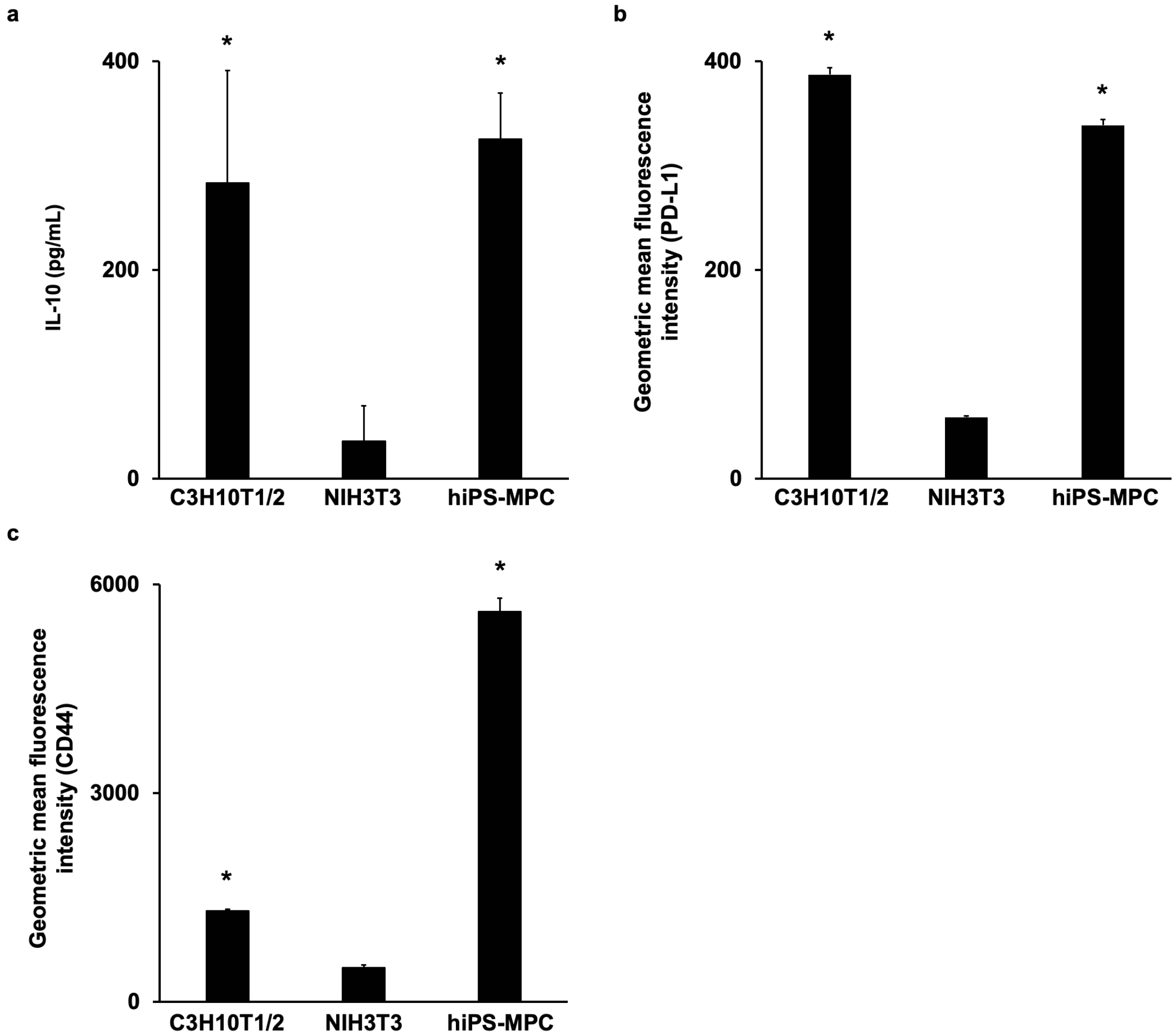

Supplementary Figure S3. Immunomodulatory properties and surface marker expression in hiPS-MPCs.

(a) Production of anti-inflammatory cytokine IL-10 by C3H10T1/2, NIH3T3, and hiPS-MPC sheets. Data are presented as mean ± standard deviation (n = 3 independent experiments). Statistical significance was determined using one-way ANOVA followed by Dunnett's multiple comparisons test. *$P < 0.05$ versus the NIH3T3 group. (b, c) Flow cytometric analysis of (b) programmed death-ligand 1 (PD-L1) and (c) CD44 expression in C3H10T1/2, NIH3T3, and hiPS-MPC sheets. Data are presented as mean ± standard deviation (n = 3 independent experiments). Statistical significance was determined using one-way ANOVA followed by Dunnett's multiple comparisons test. *$P < 0.05$ versus the NIH3T3 group.

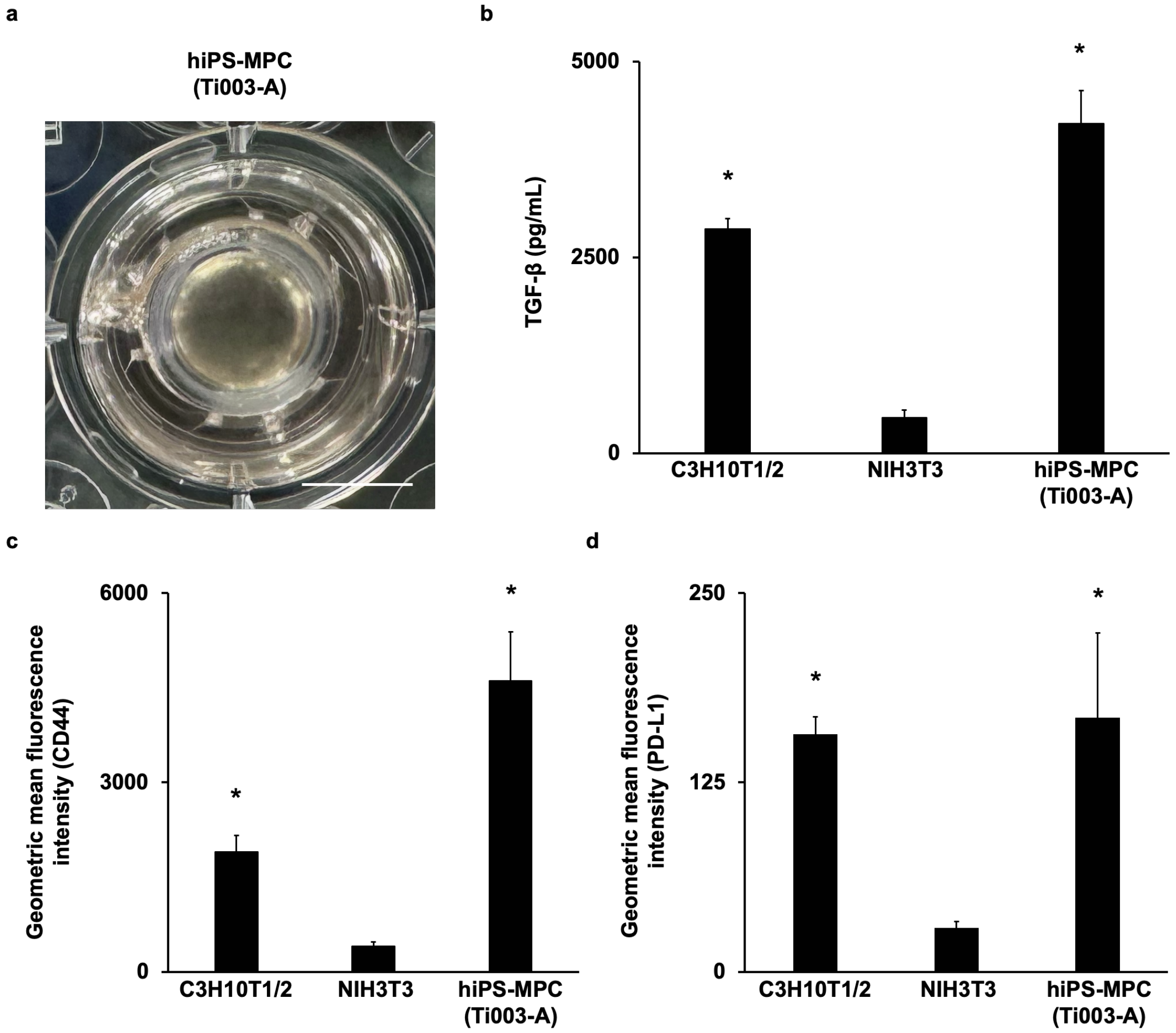

Supplementary Figure S4. Characterization of hiPS-MPCs derived from an independent hiPSC line (Ti003-A).

(a) Representative macroscopic images of cell sheets generated from hiPS-MPCs derived from independent hiPSC lines (Ti003-A) on day 5. Scale bars, 5 mm. (b) Production of anti-inflammatory cytokine TGF-β by C3H10T1/2, NIH3T3, and hiPS-MPC (Ti003-A) sheets. Data are presented as mean ± standard deviation ($n = 3$ independent experiments). Statistical significance was determined using one-way ANOVA followed by Dunnett's multiple comparisons test. *$P < 0.05$ versus the NIH3T3 group. (c, d) Flow cytometric analysis of (c) CD44 and (d) programmed death-ligand 1 (PD-L1) expression in C3H10T1/2, NIH3T3, and hiPS-MPC (Ti003-A) sheets. Data are presented as mean ± standard deviation ($n = 3$ independent experiments). Statistical significance was determined using one-way ANOVA followed by Dunnett's multiple comparisons test. *$P < 0.05$ versus the NIH3T3 group.

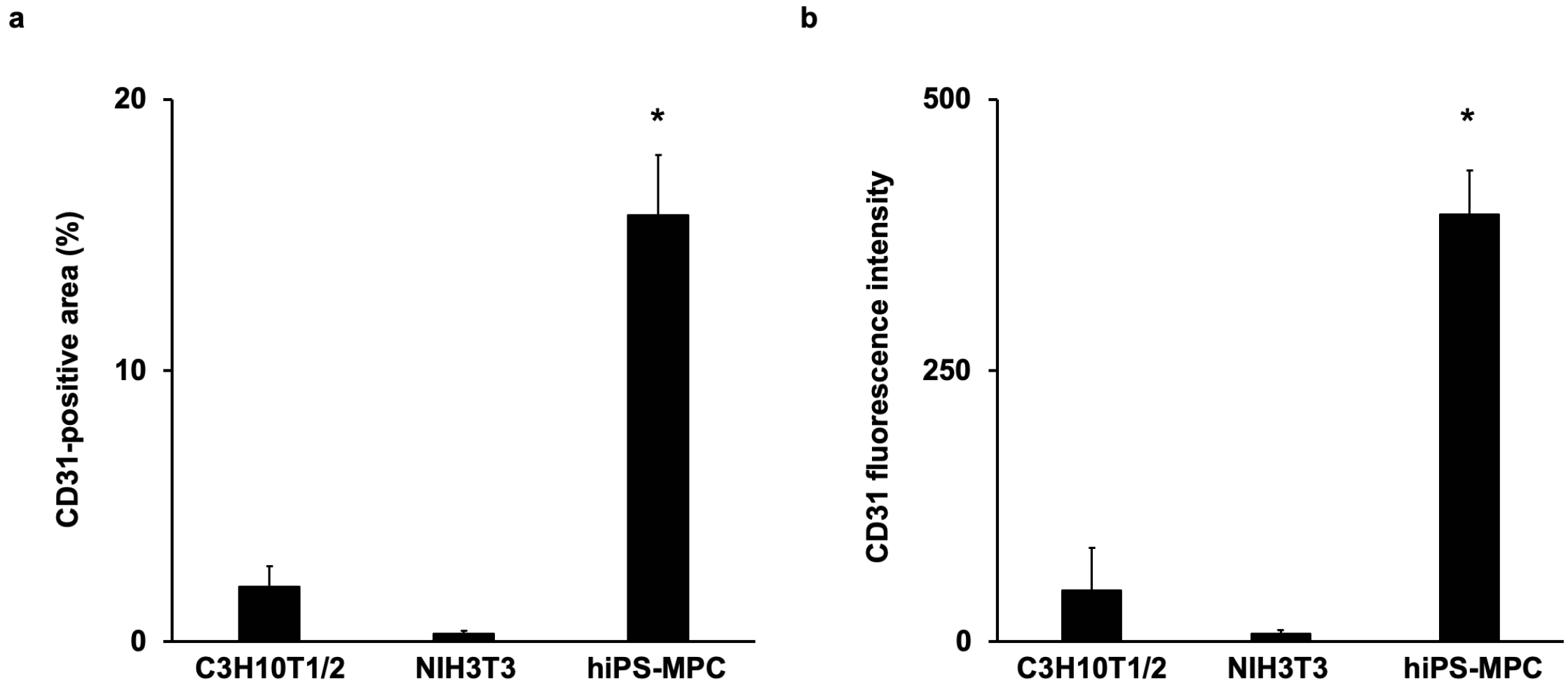

Supplementary Figure S5. Quantification of vascularization by CD31 staining.
(a) Quantification of CD31-positive area (%) is shown, indicating the extent of vascularization in each group. (b) Fluorescence intensity of CD31 staining was also quantified to assess relative expression levels. Data are presented as mean ± standard deviation ($n = 3$ independent experiments). Statistical significance was determined using one-way ANOVA followed by Dunnett's multiple comparisons test. $^*P < 0.05$ versus the NIH3T3 group.

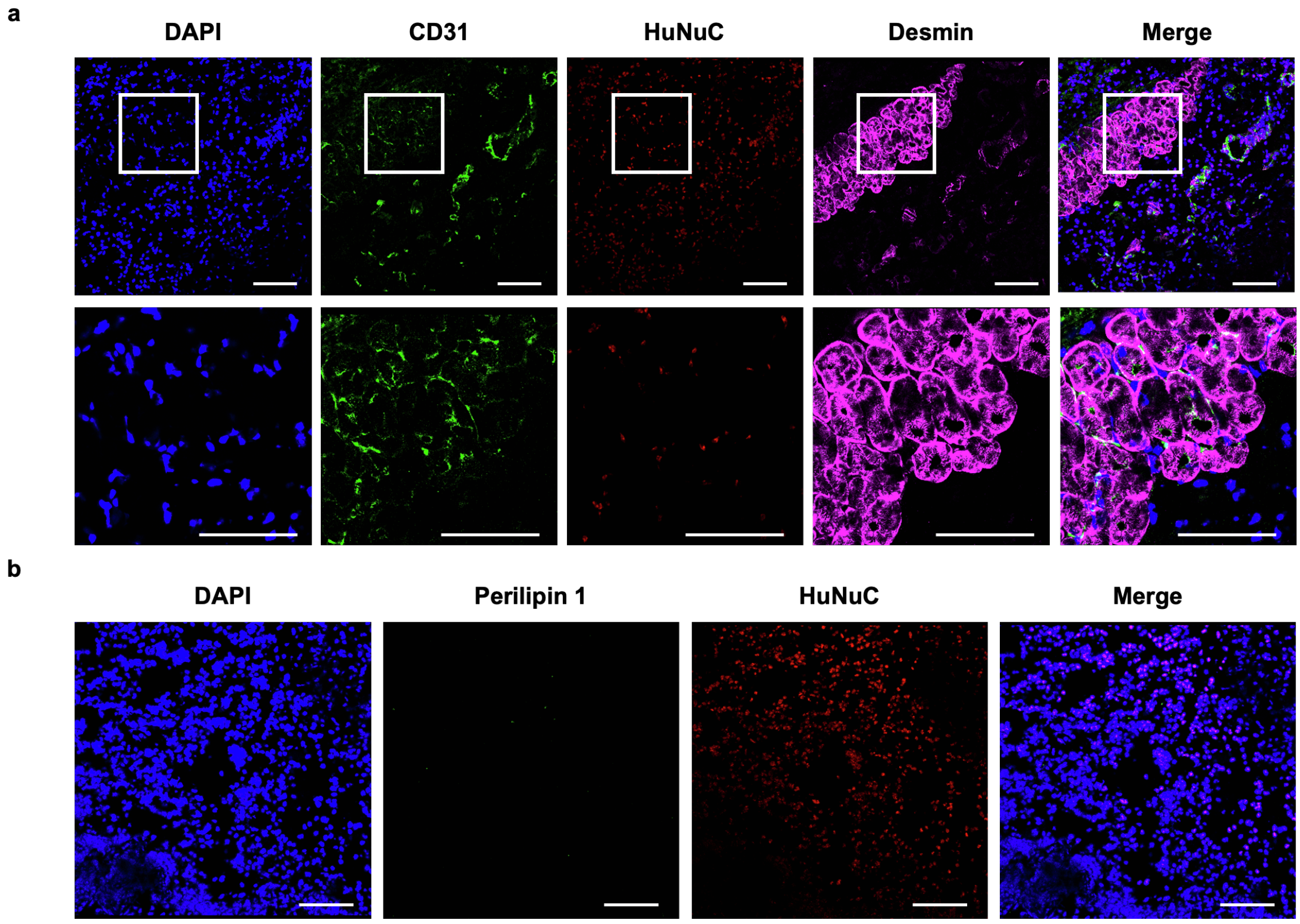

Supplementary Figure S6. UE7T-13 sheets form tissue-like structures and integrate with host tissue *in vivo*.

(a) Representative immunofluorescence images showing CD31 (endothelial cell marker), human nuclei (HuNuC), and desmin (vascular smooth muscle cell marker) in tissue-like structures formed after transplantation of UE7T-13 sheets into BALB/c mice on day 28. Blue, DAPI; green, CD31; red, HuNuC; magenta, desmin. Scale bars, 100 µm. A higher-magnification inset of the boxed region is shown to highlight luminal structures. (b) Representative immunofluorescence images showing perilipin-1 (mature adipocyte marker) and HuNuC in tissue-like structures formed after transplantation of UE7T-13 sheets into BALB/c mice on day 28. Blue, DAPI; green, perilipin-1; red, HuNuC. Scale bars, 100 µm.
